# Dynamics of *Neospora caninum*-Associated Abortions in a Dairy Sheep Flock and Results of a Test-and-Cull Control Programme

**DOI:** 10.3390/pathogens10111518

**Published:** 2021-11-20

**Authors:** Roberto Sánchez-Sánchez, Ángela Vázquez-Calvo, Mercedes Fernández-Escobar, Javier Regidor-Cerrillo, Julio Benavides, Jorge Gutiérrez, Daniel Gutiérrez-Expósito, Francisco José Crespo-Ramos, Luis Miguel Ortega-Mora, Gema Álvarez-García

**Affiliations:** 1 SALUVET Group, Animal Health Department, Faculty of Veterinary Sciences, Complutense University of Madrid, Ciudad Universitaria s/n, 28040 Madrid, Spain; robers01@ucm.es (R.S.-S.); merfer02@ucm.es (M.F.-E.); luis.ortega@vet.ucm.es (L.M.O.-M.); 2 SALUVET-Innova, S.L., Complutense University of Madrid, 28040 Madrid, Spain; avazquez@cbm.csic.es (Á.V.-C.); jregidor.saluvetinnova@gmail.com (J.R.-C.); 3 Instituto de Ganadería de Montaña (CSIC-Universidad de León), 24346 León, Spain; julio.benavides@csic.es (J.B.); dgute@unileon.es (D.G.-E.); 4 MSD Animal Health, Polígono Industrial El Montalvo, C/Zeppelin, 6—Parcela 38, Carbajosa de La Sagrada, 37008 Salamanca, Spain; jorge.gutierrez3@merck.com; 5 Consorcio de Promoción del Ovino, Camino Canillas s/n, Villalpando, 49630 Zamora, Spain; crespo@lechedeoveja.com

**Keywords:** *Neospora caninum*, abortion, sheep, transmission route, control programme

## Abstract

*Neospora caninum* is an apicomplexan parasite that can cause abortions and perinatal mortality in sheep. Although ovine neosporosis has been described worldwide, there is a lack of information about the relationship between *N. caninum* serostatus and the reproductive performance. In this study, we described the infection dynamics in a dairy sheep flock with an abortion rate up to 25% and a *N. caninum* seroprevalence of 32%. Abortions were recorded in 36% and 9% of seropositive and seronegative sheep, respectively. Seropositive sheep were more likely to abort twice (OR = 4.44) or three or more times (OR = 10.13) than seronegative sheep. Endogenous transplacental transmission was the main route of transmission since 86% of seropositive sheep had seropositive offspring. Within dams that had any abortion, seropositive sheep were more likely than seronegative ones to have female descendants that aborted (OR = 8.12). The slight increase in seropositivity with the age, the low percentage of animals with postnatal seroconversion or with low avidity antibodies, and the seropositivity of one flock dog, indicated that horizontal transmission might have some relevance in this flock. A control programme based on selective culling of seropositive sheep and replacement with seronegative animals was effective in reducing the abortion rate to 7.2%.

## 1. Introduction

*Neospora caninum* is an intracellular apicomplexan parasite with a facultative heteroxenous life cycle. Cattle, sheep, and other ungulates act as intermediate hosts, while dogs act as definitive hosts in the domestic life cycle [1]. Infection of domestic ruminants can occur postnatally through the ingestion of food and water contaminated with oocysts shed by a dog (horizontal transmission) or can take place in utero by transplacental passage of tachyzoites (vertical transmission) from the dam to the foetus during gestation. Two types of transplacental infection have been described: (i) exogenous transplacental infection, which occurs when the dam becomes infected during pregnancy; and (ii) endogenous transplacental infection, which occurs after reactivation of a pre-existing chronic infection in the dam [2].

Neosporosis is one of the most important infectious causes of abortion in cattle worldwide, with estimated annual losses of $1.298 billion in the cattle industry [3]. *Neospora caninum* was also described as a cause of reproductive disorders in sheep in 1990 [4], and the findings in the last three decades suggest that ovine neosporosis may be a relevant cause of abortions [5,6,7] or stillbirths [8,9,10] in some sheep production systems. However, the impact of *N. caninum* infection in the ovine industry should be quantified. Ovine neosporosis has been described worldwide, and the global seroprevalence ranges from 2% to 36% [1,11]. The occurrence of abortions [12,13] and stillbirths [14] have been statistically associated with *N. caninum* infection in sheep. The presence of dogs [13], the consumption of placental tissues by dogs [15], and the rearing system [16] have been identified as main risk factors for *N. caninum* infection in ovine flocks.

It was reported that 35–40% of *N. caninum* seropositive sheep in a flock suffered from reproductive failure [8]. Other studies reported that the detection rates of *N. caninum* DNA in aborted foetuses ranged from 7% to 20% [17,18,19,20]. Recently, it was demonstrated that recrudescence of a chronic infection and the subsequent endogenous transplacental transmission, the most frequent route of transmission in cattle, are also highly efficient in sheep [9]. In most of these investigations, individual reproductive data of all animals in the flocks were not available, and therefore, information about the relationship between *N. caninum* serostatus and reproductive performance is scarce [8].

Similar to cattle, no vaccine or drugs are currently licenced for the prevention of ovine neosporosis [21,22]. Control measures should aim to avoid exogenous and endogenous transplacental transmission. In cattle, test and cull strategies have been shown to be effective, but whether this is a viable economic alternative depends on the within-herd seroprevalence [23]. In contrast, there are few studies on the effectiveness of the test and cull strategy in ovine neosporosis [8]. The aims of the present study were to investigate the epidemiological characteristics of a *Neospora*-associated abortion problem in a dairy sheep flock and to evaluate the efficacy of a tailored control programme based on progressive selective culling of seropositive dams and replacement with seronegative offspring.

## 2. Results

### 2.1. NcSALUVET ELISA: Accurate Serological Test for Naturally N. caninum-Infected Sheep

**Figure 1:**
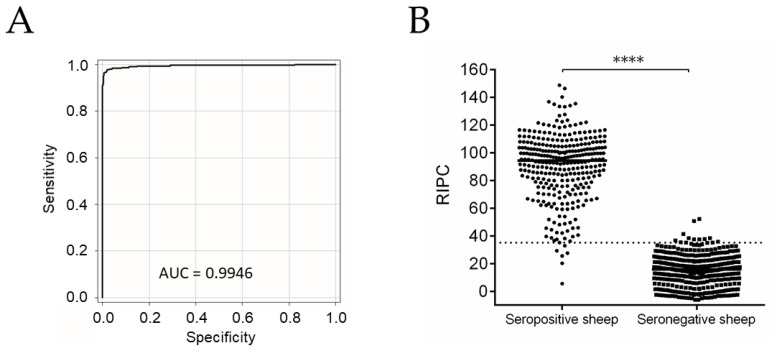
Diagnostic performance of NcSALUVET ELISA. (**A**) ROC curve for NcSALUVET ELISA. (**B**) RIPC values obtained by NcSALUVET ELISA corresponding to seropositive and seronegative sheep using coinciding results of commercial ELISA ID Screen^®^ *Neospora caninum* Indirect (IDvet, Grabels, France) and WB as reference criterion. RIPC = Relative index percent. Continuous black line in (**A**) indicates RIPC. Dotted line in (**B**) indicates cut-off (RIPC = 35.08) for NcSALUVET ELISA. For significant differences, (****) indicates *p* < 0.0001.

The optimal cut-off was estimated to be a relative index percent (RIPC) of 35.08, with 97.73% sensitivity (Se) and 97.30% specificity (Sp) (Table S1). The area under the curve (AUC) was 0.9946 (Figure 1A). Differences between seropositive and seronegative samples are graphically shown in Figure 1B. The median RIPC in the seronegative group was 15.70 (10.38–21.53 RIPC corresponding to the 25th–75th percentiles), while the median RIPC in the seropositive group was 94.35 (76.95–105.2 RIPC as the 25th–75th percentiles). Statistically significant differences were found between the seropositive and seronegative groups (*p* < 0.0001). Next, the standardized NcSALUVET ELISA for sheep sera was employed to determine *N. caninum* intraflock seroprevalence and to follow up the control programme.

### 2.2. Neospora caninum Infection Was Widespread and a Major Cause of Abortion in the Flock

#### 2.2.1. *Neospora caninum* DNA and Histological Lesions Were Detected in Placentas and Foetuses from Aborted Sheep

Eight of sixteen (50%) collected placentas and 15 of 39 (38.5%) aborted foetuses were positive for *N. caninum* DNA presence by PCR. All aborted dams with *N. caninum* PCR-positive placentas or with *N. caninum* PCR-positive aborted foetuses were *N. caninum* seropositive. By contrast, *Toxoplasma gondii* was ruled out as a major cause of abortions since *T. gondii* DNA was only detected in 1 out of 39 (2.6%) of the aborted foetuses analysed. In addition, only 18 out of 160 (11.2%) aborted sheep were seropositive to *T. gondii* by TgSALUVET ELISA. All *T. gondii* seropositive sheep were born before December 2016, and none had been vaccinated against toxoplasmosis (only animals used for replacement from 2017 onward were vaccinated).

**Figure 2:**
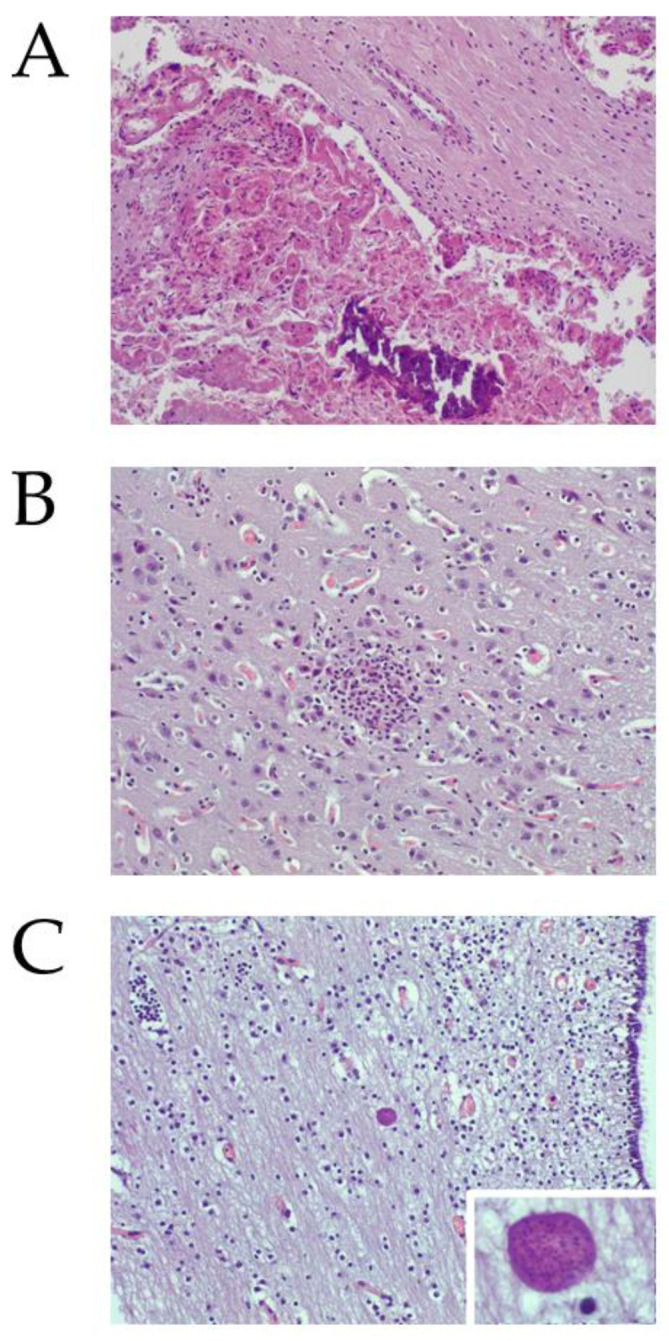
Histological findings in the placenta (**A**) and foetal brain (**B**,**C**) from aborted animals. (**A**) Necrosis of the foetal villus with an intralesional area of mineralization (lower side of the picture). Epithelial cells of the villus are shown. There is sloughing of necrotic cells and cellular debris from the epithelial cell layer into the foetal–maternal interface. The cells still attached to the villus show shrinkage, eosinophilic cytoplasm, and pyknotic nuclei. Notice the absence of evident inflammatory reaction at the maternal stalk (upper side of the picture). HE: 20×. (**B**) Mild non-suppurative encephalitis. In the neuroparenchyma of the grey matter, at the hypothalamus level, there was a well-delimitated glial focus. HE: 20×. (**C**) Tissue cyst-like structure. The tissue cyst-like structure was located in the white matter of the hypothalamus, at the periventricular area. HE: 20×. Inset: High power magnification of the tissue cyst-like structure. HE: 60×.

Tissue was too damaged in 6 of the 16 placentas and in 7 of the 39 brains from aborted foetuses to allow for proper histological examination. Characteristic lesions of protozoal abortion were found in the studied samples. Nine of ten (90%) examined placentas showed multifocal necrotic foci (Figure 2A). In the brains from aborted foetuses, mild non-suppurative encephalitis, denoted by glial foci, were found in 14 of the 32 (43.8%) foetal brains studied (Figure 2B). In addition, several brains from aborted foetuses showed parasite structures consistent with tissue cysts (Figure 2C). These structures were found in normal neuroparenchyma and in areas with histological lesions. Within the thirteen *N. caninum* PCR-positive brains in which histological evaluation was carried out, nine (69%) had lesions. On the other hand, within the nineteen *N. caninum* PCR-negative in which histological evaluation was carried out, five (26.3%) had lesions. 

#### 2.2.2. Abortions Were Associated with *N. caninum* Infection

**Figure 3:**
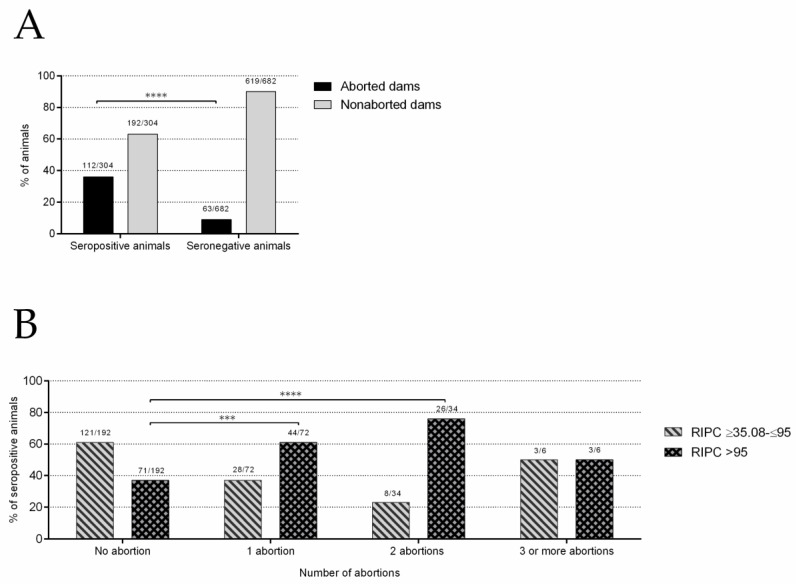
Association between *N. caninum* seropositivity and abortion. (**A**) Abortion rates in seropositive and seronegative sheep. (**B**) Number of abortions in seropositive sheep according to anti-*N. caninum* IgG levels (RIPC values). For significant differences, (***) indicates *p*< 0.001 and (****) indicates *p* < 0.0001.

Of 986 female sheep, 304 were seropositive to *N. caninum* by NcSALUVET ELISA (apparent flock seroprevalence rate of 30.8%). The true flock seroprevalence rate was 32.4% (95% confidence interval: 27.7–37.2%). Abortions were recorded in 36.8% and 9.2% of seropositive and seronegative sheep, respectively. Seropositive dams were 5.73 times more likely to abort than seronegative ones (*p* < 0.0001, odds ratio (OR) = 5.73) (Figure 3A).

#### 2.2.3. *Neospora caninum* Seropositive Sheep Are More Likely to Have Repeated Abortions

At the sampling time, within seropositive sheep that had aborted (*n* = 112), one abortion was recorded in 64.3% (72/112), two abortions were recorded in 30.4% (34/112), and three or more abortions were recorded in 5.3% (6/112). On the other hand, within seronegative sheep that had aborted (*n* = 63), one abortion was recorded in 88.9% (56/63) and two abortions were recorded in 11.1% (7/63). Accordingly, seropositive animals were more likely to abort twice (*p* < 0.001; OR = 4.44) or three or more times (*p* < 0.05; OR = 10.13) than seronegative animals.

Comparing the likelihood of abortion in seropositive animals according to the RIPC values, animals with RIPC values higher than 95 (47.3% of the seropositive animals) were more likely to abort than animals with RIPC values between 35.08 and 95 (*p* < 0.0001; OR = 3.19). In more detail, animals with RIPC values between 75 and 95 tended to have more abortions than animals with RIPC values between 55 and 75 (*p* < 0.05; OR = 2.92). Comparing the number of abortions, animals with RIPC values higher than 95 were more likely to have one abortion (*p* < 0.001; OR = 2.67) or two abortions (*p* < 0.0001; OR = 5.53) than animals with RIPC values between 35.08 and 95 (Figure 3B).

### 2.3. Neospora caninum Genotyping Suggests a Unique Source of Infection in the Flock Maintained by Clonal Propagation

Complete or almost complete microsatellite genotypes were obtained for 7 of 15 brains collected from aborted foetuses (foetuses 1, 4, 5, 9, 12, 13, and 14) (Table 1). A predominant microsatellite genotype was identified across all foetal brain PCR-positive samples with three variants that only differed in a single repeating unit in the MS5, MS8, or MS10 loci. One genotype included both identified alleles for MS5, likely as a consequence of microsatellites in the status of transition instead of a mixed infection.

### 2.4. Neospora caninum Endogenous Transplacental Transmission Was Predominant in the Flock

A total of 74 of the 245 dams (in which dam–offspring pairs were established) were seropositive. A total of 64 of the 74 seropositive sheep (86.5%) had seropositive female descendants. More specifically, fifty-five sheep (74.3%) showed 100% seropositive female descendants, and nine (12.2%) had 50% seropositive female descendants (one of two female descendants available in the flock were seropositive). The remaining seropositive dams had no seropositive female descendants (*n* = 10; 13.5%). *Neospora caninum* seropositive sheep were more likely to have seropositive descendants than seronegative sheep (*p* < 0.0001; OR = 58.55).

**Figure 4:**
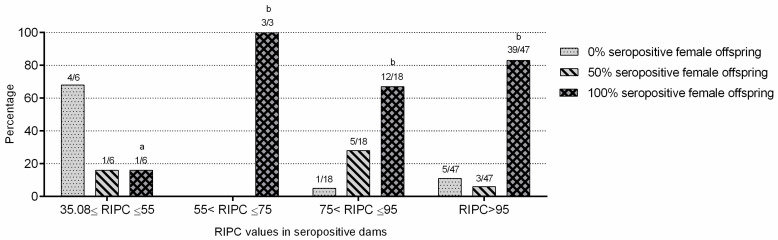
*N. caninum* seropositive dam–offspring comparisons. Different letters (a,b) indicate statistically significant differences. 35.08 ≤ RIPC ≤ 55 vs. 55< RIPC ≤ 75, *p* < 0.05; 35.08 ≤ RIPC ≤ 55 vs. 75 < RIPC ≤ 95, *p* < 0.05; and 35.08 ≤ RIPC ≤ 55 vs. RIPC > 95, *p* < 0.01.

Seropositive sheep with RIPC values higher than 95 were more likely to have 100% seropositive female offspring than seropositive sheep with lower RIPC values (*p* < 0.05; OR = 3.35). Additionally, seropositive sheep with RIPC values between 35.08 and 55 were significantly less likely to have 100% seropositive female offspring than those with RIPC values higher than 55 (Figure 4). When the presence or absence of abortion in seropositive dams was compared with the percentage of seropositive female offspring (0, 50, and 100%), no statistically significant differences were found; thus, seropositive and aborted dams had no more percentage of seropositive female offspring than seropositive and non-aborted ones.

*N. caninum* seropositive and aborted dams were more likely to have offspring that aborted than *N. caninum* seronegative and aborted dams (*p* < 0.0001; OR = 8.12) (Table 2).

However, no significant differences in the occurrence of abortion were observed between the offspring born from seropositive and seronegative non-aborted dams.

### 2.5. Horizontal Transmission Might also Contribute to a Low Percentage of Infections

**Figure 5:**
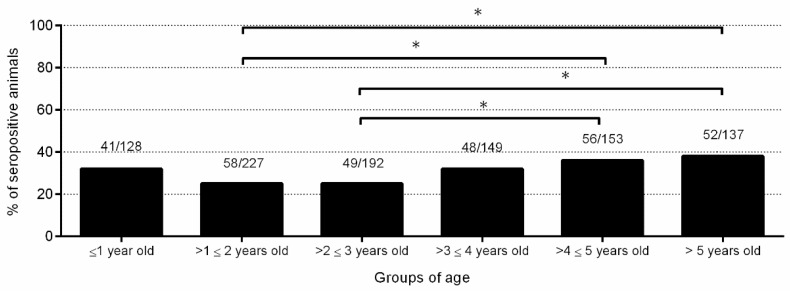
Seroprevalence rates in different age groups. For significant differences, (*) indicates *p* < 0.05.

A significantly higher *N. caninum* seroprevalence rate was found in sheep between 4 and 5 years old (36.6%) and older than 5 years (38%) than in sheep between 1 and 2 years old (25.6%) and between 2 and 3 years old (25.5%) (*p* < 0.05) (Figure 5). Eight of eight (100%) serum samples from seropositive and aborted sheep, collected one month after abortion, showed a high avidity index (AI) (54.7 ± 13.6). In addition, four of six seropositive non-aborted sheep (66.6%) showed a high AI (48 ± 7.1), one (16.7%) showed an intermediate AI (29.8), and one (16.7%) showed a low AI (19.7).

Regarding dam–offspring pairs, 19 of 340 (5.6%) female offspring born from *N. caninum* seronegative dams were *N. caninum* seropositive. Ten (52.6%) seropositive female offspring showed RIPC values between 35.08 and 55, and three of them (30%) aborted once. Three (15.8%) seropositive female offspring showed RIPC values between 55 and 95, and one of them (33.3%) aborted once. Six (31.6%) seropositive female offspring showed RIPC values higher than 95, and two of them (33.3%) aborted twice.

When offspring were resampled, 8 of the 278 (2.9%) seronegative female lambs at one month of age were seropositive at 7–10 months of age, with RIPC values of 47 ± 17.1. No reproductive data of these eight animals that seroconverted were available since they were culled shortly after seroconversion. Regarding the flock dogs, 1 of the 2 dogs was seropositive to *N. caninum* with a titre of 1:50 by indirect fluorescence antibody test (IFAT).

### 2.6. Progressive Selective Culling of N. caninum Seropositive Animals and Replacement with Seronegative Animals Reduced Abortion Rate in the Flock

Among 304 seropositive animals, 222 (73%) were culled, with 126 (41.4%) and 96 (31.6%) seropositive animals culled in 2018 and 2019, respectively. A total of 85.4%, 71.8%, 54%, and 45.9% of animals with RIPC values higher than 95, between 75 and 95, between 55 and 75, and between 35.08 and 55, respectively, were culled. During the control programme, replacement was carried out with seronegative female sheep born from *N. caninum* seronegative dams.

Compared to the abortion rate in 2018 (25.6%), a significant reduction in the abortion rate was found upon implementation of the control programme in 2019 (16.3%; *p* < 0.0001) and 2020 (7.2%; *p* < 0.0001). Additionally, a significant reduction was found in the abortion rate between 2019 and 2020 (*p* < 0.0001) (Table 3).

## 3. Discussion

Herein, the dynamics of *N. caninum*-associated abortions in a dairy sheep flock were described. First, *N. caninum* was confirmed as the main cause of the high abortion rate. Then, endogenous transplacental transmission was identified as the predominant route of parasite transmission. Based on these observations, a flock-specific control programme was implemented and proved to be effective.

Although seroprevalence studies indicate that *N. caninum* infection is present in small ruminants worldwide [1,15,25,26], the impact of ovine neosporosis is still unknown, and only a few case reports of *N. caninum* associated abortions have been published [5,6,7]. On the other hand, previous experiences of controlling ovine neosporosis are scarce [8]. *Neospora caninum* serological diagnosis is a key tool to monitor the infection dynamics and follow up the control programme in ovine flocks. There are a few commercial ELISAs adapted for sheep sera, but no ring trials showing comparable results among them have been carried out. Therefore, a soluble tachyzoite antigen-based ELISA (NcSALUVET ELISA) was standardized for sheep sera. This novel assay proved to be accurate with Se and Sp values higher than 97%, so it was employed in this study.

*Neospora caninum*-associated abortions proved to be a major problem in this flock. Abortions are a major source of economic losses in small ruminant flocks [27]. Traditionally, the zoonotic parasite *T. gondii* was considered as the main parasite causing abortion in sheep [28]. However, recent evidence suggests that *N. caninum* is also linked to abortion and neonatal mortality in sheep [5,7,8,10]. Most of the studies focused on *N. caninum* in sheep have investigated the seroprevalence by sampling a representative number of animals from several flocks [13,15,29,30] or testing for *N. caninum* DNA presence in aborted foetuses from several flocks [17,20,31,32]. However, only one study addressed the relationship between *N. caninum* serostatus and reproductive performance in a sheep flock [8]. In Spanish sheep flocks, the seroprevalence of *N. caninum* ranged from 1.9% to 10.1% [33,34,35,36]. However, in flocks with reproductive failure associated with *N. caninum* infection, the seroprevalence was higher (30–34%) [8,10] and similar to the seroprevalence rate (32%) found in this study. Moreover, the histological lesions compatible with *N. caninum* infection and the high *N. caninum* DNA detection rate found in placentas and aborted foetuses from seropositive and aborted dams corroborated that *N. caninum* was a major cause of abortions in the investigated flock. In contrast, *T. gondii* DNA was only detected in 2.6% of the brains collected from the aborted foetuses and only 11.2% of the aborted sheep were seropositive to *T. gondii*. This stands in contrast to previous studies where *T. gondii* was a relevant cause of ovine abortions [37,38].

The abortion rate in this flock reached 25.6% in 2018, which is far above the 5% considered acceptable [39]. An abortion rate higher than 5% was previously described in ovine flocks with *N. caninum*-associated abortions [5,10]. The presence of stillbirths was registered; however, contrary to previous studies [8,9,10], stillbirths were not differentiated from foetal losses during pregnancy. Ovine and bovine neosporosis shared several similarities such as the occurrence of repeated abortions and its association with high antibody titres and the predominance of efficient endogenous transplacental transmission. In this flock, repeated abortions were common and were associated with *N. caninum* infection. Repetitive abortions have been previously described in sheep with natural *N. caninum* infections [5,7] and under experimental conditions [40]. Seropositive sheep were 5.7 times more likely to abort than seronegative sheep, and 36.8% of *N. caninum* seropositive dams aborted, similar to previous reports [6,8,9] that also found a significant association between *N. caninum* seropositivity and abortion. Moreover, this is the first study that describes an association between high antibody titres and repetitive abortions in sheep as previously reported for cattle [41]. Thus, individual *N. caninum*-specific antibody levels (and not only seropositivity) can be used as predictors of abortion in sheep flocks with a high *N. caninum* seroprevalence rate.

As in cattle, endogenous transplacental transmission (due to reactivation of latent *N. caninum* infections during pregnancy) has been found to be highly successful in sheep (96.6% of gestations) [9] and in goats (71.4–100% vertical transmission rate) [42,43]. However, other studies in sheep described low vertical transmission rates (15–31%) without associated abortions [44,45]. In this flock, endogenous transplacental transmission was the main route of parasite transmission since 86.5% of the offspring born from *N. caninum* seropositive sheep were seropositive. Similarly, a 93% endogenous transplacental transmission rate was described in another sheep flock in Spain [8]. As in previous studies carried out in cattle and sheep [45,46,47], we also found that seropositive sheep with high antibody titres were more likely to have seropositive offspring. Thus, individual *N. caninum*-specific antibody levels can also be used as a predictive tool for identifying animals with a high risk of endogenous transplacental transmission. In addition, *N. caninum* seropositive aborted dams were more likely to have offspring that aborted. The presence of abortion in the dams and in their offspring had been previously found in cattle [48]; however, a statistical association was not confirmed. In this flock, the practice of self-replacement regardless of their serostatus was likely the cause of the increasing abortion rate over time. In the same way, the identification of a single genotype by microsatellite genotyping suggests the clonal propagation of *N. caninum* in the flock and the entry of disease in the flock from a unique source of infection. The genotype found is very similar to that described in another sheep flock in Spain [8,9,49], with variations only in MS4, MS5, and MS21 markers.

Regardless of the importance of endogenous transplacental transmission, horizontal transmission could have also contributed to a low number of infections. First, an increased seropositivity rate with age was found. Second, although in this flock most of the analysed seropositive animals showed a high AI (indicating a chronic infection), there was one sheep with an intermediate AI and one sheep with a low AI, which may indicate a recent postnatal infection [50]. Moreover, a low percentage of animals (5.6%) born from seronegative sheep seroconverted postnatally and showed low antibody titres, as in a previous study [8], which could indicate a postnatal infection [51]. The serological results of the flock dogs living alongside the sheep (one of them seropositive) are not conclusive for either endogenous transplacental or postnatal transmission. Dogs can disseminate *N. caninum* oocysts in the flock, although the presence of anti-*N. caninum* antibodies in dogs does not necessarily indicate environmental contamination. In addition, the majority of dogs that shed *N. caninum* oocysts did not show seroconversion [1].

A policy of test and cull can be an efficacious strategy in the short term to reduce *N. caninum* infection (and the associated reproductive failure) in a flock with a clear predominance of endogenous transplacental transmission [8]. To implement an economically viable control programme in this flock, progressive culling of 73% of the seropositive animals (prioritizing the animals with high antibody titres, which were also the ones with a higher number of abortions and seropositive offspring) was carried out during 2018 and 2019, along with replacement with seronegative animals born from seronegative sheep. The abortion rate in the flock showed a significant decrease upon implementation of the control programme, decreasing from 25.6% in 2018 to 16.3% and 7.2% in 2019 and 2020, respectively. However, the abortion rate at the end of this study (7.2%) was still higher than 5%, probably because of the maintenance of seropositive sheep in the flock (some of them with high antibody levels) but possibly due also to the presence of other abortifacient agents. The replacement policy with a first serological diagnosis of the female offspring at one month of age, when colostral antibodies decrease more than 50% [52] (to have the chance to cull the lambs for meat production if they are seropositive) and a second testing at 7–10 months of age (before sexual maturity), proved to be a rational strategy because it permitted the detection of seroconversions and only mate seronegative animals.

In conclusion, *N. caninum* infection dynamics in this dairy sheep flock were characterized by a high abortion rate, the occurrence of repetitive abortions, and the predominance of endogenous transplacental transmission with a low contribution of the horizontal transmission route. Serological diagnosis was a key tool to determine the relevance of *N. caninum* infection and the main route of transmission, but also to establish the association between infection and abortion. Moreover, serology was also very useful to implement a control programme based on test, selective, and progressive culling and replacement with seronegative offspring. This strategy significantly reduced the impact of *N. caninum* infection in the flock in the short term.

## 4. Materials and Methods

### 4.1. Flock Description and Collection of Data

A semi-intensive pure Assaf breed dairy ovine flock located in the province of Zamora (Spain) was examined. The animals were fed a balanced ration (unifeed) containing concentrate (corn, barley, soy, beet pulp, and corn distiller’s grains) and forage (oat hay, wheat hay, and corn silage). As part of the health programme, vaccines against the following transmissible diseases were used: chlamydiosis and salmonellosis (Inmeva^®^, Hipra, Girona, Spain), border disease (Bovilis BVD^®^, MSD, Salamanca, Spain, only in 2017), toxoplasmosis (Ovilis Toxovac^®^, MSD, Salamanca, Spain, only in animals used for replacement from 2017 onwards), clostridial diseases (Syva-Bax^®^, Syva, León, Spain), contagious agalactia (Algontex^®^, Vetia Animal Health, Pontevedra, Spain), staphylococcal mastitis (Vimco^®^, Hipra, Girona, Spain), and respiratory diseases (Ovipast Plus^®^, MSD, Salamanca, Spain). Two 6- and 9-year-old mixed breed dogs were present in the flock. The reproductive programme of the flock was scheduled in four lambing periods per year. Rams were used to mate female sheep, which had natural heat during the oestrus period and hormonally induced heat during the anoestrus period (from February to September).

Data from all the animals, including ascendants and/or descendants in the flock, ages and dates of lambing (or abortion) and, if necessary, culling date, were recorded in Microsoft Excel^®^ from 2011 to 2020. Abortion was defined as a termination of pregnancy between days 40 and 150 of gestation, including stillbirths. The number of female adult sheep and the percentages of abortion, culling, and replacement from 2011 to 2020 (before and during the control programme) are stated in Table 3. The abortion rate (number of animals with abortion/total number of animals) showed a steady increase from 8.11% in 2011 to 25.64% in 2018. In 2017, border disease virus was detected by PCR in one aborted foetus, and no PCR-positive results for *Coxiella burnetii* and *Chlamydia abortus* were obtained in three placentas (unpublished data). The culling rate (number of culled animals/total number of animals) increased from 11.2% in 2011 to 40.6% in 2018. The replacement rate (number of animals replaced/total number of animals) was also high and increased over time (from 14% in 2011 to 34.8% in 2018) (Table 3). Data from 245 dams with 449 female offspring used for replacement in the flock (245 dam–offspring matches) were used to investigate the relationship between the seropositivity and abortion of dams and their female offspring.

### 4.2. Samplings

The collection of samples and the techniques employed are summarized in Table 4. From March to May 2018, blood samples were collected from 986 female sheep (862 adult female sheep and 125 young female replacement animals) via jugular venipuncture into 5 mL vacutainer tubes without anticoagulant (Becton Dickinson and Company, Plymouth, UK). In March 2018, blood samples were collected from the two dogs present in the flock by venipuncture of the cephalic vein into 5 mL vacutainer tubes without anticoagulant (Becton Dickinson and Company, Plymouth, UK). All blood samples were sent to SALUVET-Innova S.L. within 24 h after collection. Vacutainer tubes were allowed to clot and were centrifuged at 450× *g* for 10 min at 4 °C to obtain serum samples that were stored at −80 °C until serological analysis. Additionally, of the 221 sheep aborted in 2018, 39 aborted foetuses (from 34 sheep) and 16 placentas (from another 16 aborted sheep) were collected and kept frozen at −20 °C in the flock facilities until submission for further examination. After necropsy at the Instituto de Ganadería de Montaña (CSIC-University of León), cotyledons and foetal brain samples were taken for histological and molecular examination.

From June 2018 and during 2019 and 2020, blood samples were collected from 972 female lambs born from *N. caninum* seronegative dams (which were candidates for replacements of the flock during the control programme) at one month of age by jugular venipuncture into 5 mL vacutainer tubes without anticoagulant (Becton Dickinson and Company, Plymouth, UK). A total of 278 of these 972 female lambs were resampled between 7–10 months of age. The blood samples were sent to SALUVET-Innova S.L. and were processed and stored as previously stated for the blood samples collected in early 2018.

### 4.3. Parasites

Tachyzoites needed for ELISA and PCR techniques were obtained in cell culture. *Neospora caninum* (Nc-Spain7 isolate) and *T. gondii* (TgME49 isolate) tachyzoites were propagated by continuous passage in MARC-145 cells. The parasites were harvested from tissue culture and purified by a PD-10 column (Cytiva, Little Chalfont, UK) [53]. Purified tachyzoites were pelleted and stored at –80 °C until use.

### 4.4. Serological Assays

#### 4.4.1. NcSALUVET ELISA

Sheep sera were tested for *N. caninum* IgG by an in-house indirect enzyme-linked immunosorbent assay (named NcSALUVET ELISA) that employed soluble *N. caninum* tachyzoite extract as antigen. NcSALUVET ELISA was carried out as previously described [54] but with the following modifications: (i) plates were blocked with 1% bovine serum albumin instead of 3% bovine serum albumin, and (ii) horseradish peroxidase-conjugated monoclonal anti-goat/sheep IgG (A9452-Sigma-Aldrich, Madrid, Spain) diluted 1:20,000 in PBS (pH 7.4) containing 0.05% Tween 20 (PBS-T) was used instead of horseradish peroxidase-conjugated protein G. One serum from an *N. caninum* experimentally infected sheep and one serum from a non-infected sheep were used as controls [54]. The optical density (OD) was read at 405 nm (OD405), and the reaction was stopped when the OD405 of the positive control = 1 by the addition of 100 μL/well of 0.3 M oxalic acid. The OD405 values were converted into RIPC using the formula RIPC = (OD405 sample—OD405 negative control)/(OD405 positive control—OD405 negative control) × 100.

To determine the diagnostic performance of the NcSALUVET ELISA adapted to sheep serum samples, receiver operating characteristic (ROC) analysis was performed with 791 ovine serum samples from the flock studied herein (309 *N. caninum* seropositive and 482 *N. caninum* seronegative serum samples). To determine sample serostatus by NcSALUVET ELISA, coinciding results of the commercial ELISA ID Screen^®^ *Neospora caninum* Indirect (IDvet, Grabels, France) (see Section 4.4.2) and WB (see Section 4.4.3) were considered as the reference.

#### 4.4.2. ID Screen^®^ *Neospora caninum* Indirect ELISA

The commercial ID Screen^®^ *Neospora caninum* Indirect ELISA was performed according to the manufacturer’s instructions. OD was read at 450 nm, and S/P% was calculated following the formula S/P% = (OD sample—OD negative control)/(OD positive control—OD negative control) × 100. Samples with S/P% >40 and <50 were considered doubtful, and samples with S/P% ≥ 50 were considered positive.

#### 4.4.3. Western Blot

Western blotting was carried out as previously described [55]. *Neospora caninum* tachyzoites were electrophoresed under reducing conditions (2 × 10^7^ tachyzoites per membrane). Serum samples were 1:20 diluted. A positive result was considered when three of the four immunodominant tachyzoite antigens (17–18, 34–35, 37, and 60–62 kDa antigens) were recognized [55].

#### 4.4.4. NcSALUVET Avidity ELISA

Eight seropositive sera collected one month after abortion and six sera from seropositive animals without a previous history of abortions were analysed by NcSALUVET avidity ELISA. NcSALUVET avidity ELISA was carried out as previously described [56]. Briefly, sera were tested using duplicate 4-fold dilution series starting at 1:25 up to 1:25,600. After 1 h of incubation at 37 °C, one dilution series was washed with washing solution, and the other was washed 3 times with 6 M urea diluted in PBS-T. The next steps were basically those described above for NcSALUVET ELISA. IgG avidity was expressed as an avidity index (AI). AIs lower than 25%, between 25% and 40%, and above 40% were classified as low, intermediate, and high avidity, respectively.

#### 4.4.5. TgSALUVET ELISA

A total of 160 serum samples from dams that were aborted prior to the sampling date were analysed for anti-*T. gondii* IgGs using the indirect TgSALUVET ELISA [57]. This test employed *T. gondii* soluble antigens and showed a sensitivity of 98.2% and a specificity of 97.7%.

#### 4.4.6. Indirect Fluorescence Antibody Test (IFAT)

In dog sera, the detection of anti-*N. caninum* IgGs was carried out by IFAT as previously described [58]. Serum samples showing fluorescence at a 1:50 dilution (cut-off value) were titrated using two-fold serial dilutions until fluorescence extinction.

### 4.5. Histological Examination

After fixation for 5 days in 10% buffered formalin, the samples from the cotyledons and a piece of the foetal brain were processed at the Instituto de Ganadería de Montaña (CSIC-University of León). Sections 4 mm thick were routinely prepared and stained with haematoxylin and eosin (HE). Conventional histological evaluation was carried out in all the sections.

### 4.6. PCRs

One cotyledon of each placenta and a piece of the brain from the aborted foetuses were submitted to SALUVET-Innova S.L. and stored at −80 °C until PCR analysis. Genomic DNA was extracted from 20–50 mg of each tissue using the commercial Maxwell^®^ 16 Mouse Tail DNA Purification Kit developed for the automated Maxwell^®^ 16 System (Promega, Madison, WI, USA) following the manufacturer’s recommendations. Three different DNA extractions from each tissue were carried out. The DNA concentration of each sample was determined using a Synergy^®^ H1 multimode microplate reader (Biotek, Winooski, VT, USA) and Gen5 version 2.09.1 software (Biotek, Winooski, VT, USA) and adjusted to 100 ng/µL in RNase-, DNase-, and protease-free water.

*Neospora caninum* and *T. gondii* DNA detection was carried out by PCR amplification of the Nc5 region of *N. caninum* [59] and the ITS1 region of *T. gondii* [60]. Each PCR was performed in a final volume of 25 μL using 5 μL of genomic DNA. Positive *N. caninum* and *T. gondii* samples equivalent to 10 (*n* = 1), 1 (*n* = 3), and 0.1 (*n* = 2) tachyzoites in 100 ng of sheep genomic DNA were included in each round of DNA extraction and PCR. Additionally, negative controls from uninfected animals were included in each round of DNA extraction and PCR. Ten-microlitre aliquots of the PCR products were visualized under UV light in a 1.5% agarose gel stained with GelRed^®^ nucleic acid gel stain (Biotium, Inc., Fremont, CA, USA) to detect the *N. caninum*-specific 337 bp and the *T. gondii*-specific 227 bp amplification products. Validation of PCR assays required the absence of amplification of the sample from the uninfected animals and the amplification of at least two positive controls (10 and 1 tachyzoites).

### 4.7. Microsatellite Genotyping Method

DNA samples obtained from *N. caninum* PCR-positive foetal brains were used for genotyping by multilocus microsatellite analysis. Specifically, MS4, MS5, MS6A, MS6B, MS7, MS8, MS10, MS12, and MS21 markers were amplified using specific primers and nested PCR conditions, as previously described [24]. For all microsatellites, the size of the PCR products was determined in a 48-capillary 3730 DNA analyser (Applied Biosystems, Foster City, CA, USA) with GeneScan-500 (LIZ) size standards (Applied Biosystems) at the Genomics Unit of the Complutense University of Madrid, Spain. The results were analysed with GeneMapper1 software v3.5. To confirm allele identification, microsatellite alleles MS4, MS5, MS6A, MS7, MS10, and MS21 from representative samples were sequenced using a Big Dye Terminator v3.1 cycle sequencing kit (Applied Biosystems) and a 3730 DNA analyser (Applied Biosystems). Sequences were analysed using BioEdit Sequence Alignment Editor v.7.0.1 software (Copyright 1997–2004 Tom Hall, Ibis Therapeutics, Carlsbad, CA, USA). Allele assignment was performed as previously described [24].

### 4.8. Test and Cull Control Programme

A control programme was designed to reduce endogenous transplacental transmission without hampering the replacement of animals within the flock. Progressive selective culling of *N. caninum* seropositive animals (in 2018 and 2019, prioritizing the animals with higher RIPC values) and replacement with seronegative animals born from seronegative dams was implemented.

### 4.9. Data Analysis

SigmaPlot version 12.0 from Systat Software, Inc. (San Jose, CA, USA) was used to carry out the ROC analysis. The area under the curve (AUC) was used to determine the diagnostic performance of the NcSALUVET ELISA, and the optimal cut-off was selected based on the highest Se and Sp values achieved. A Mann–Whitney U-test was used for pairwise comparisons between the RIPC values in the seropositive and seronegative groups. Intraflock seroprevalence was estimated (number of seropositive animals/number of animals analysed) and adjusted to the test Se and Sp values to obtain the true prevalence [61]. Fisher’s exact test (some values with *n* ≤ 10) and OR were used to evaluate the relationship between seropositivity and the number of abortions and the RIPC values of seropositive animals and the number of abortions. A chi-square test (χ²) (values with *n* > 10) was used to evaluate significant differences between seropositivity and abortions and between the seroprevalence rates according to the age of the animals.

The relationship between the seropositivity and abortion of dams and their female offspring was investigated in 245 dams with 449 female offspring used for replacement in the flock (245 dam–offspring matches). The percentage of *N. caninum* seropositive female descendants born from an *N. caninum* seropositive dam was calculated by dividing the number of *N. caninum* seropositive female descendants by the total number of descendants available for the seropositive dam (all available generations of animals until 2018 were used). For the association between abortions in dams and in their offspring, reproductive data until 2020 were recorded. Fisher’s exact test (some values with *n* ≤ 10) and OR were used to evaluate the relationship between RIPC values of seropositive dams and the existence of seropositive female offspring, the percentage of seropositive female offspring born from seropositive sheep according to the presence or absence of abortion, and the seropositivity and abortion of the dams and their female offspring. The percentages of aborted animals during the control programme were compared using the chi-square test (χ²) (values with *n* > 10). All statistical analyses were performed using GraphPad Prism 6.01 software (San Diego, CA, USA). Significant differences were considered when the *p* value < 0.05.

## Figures and Tables

**Table 1:** *N. caninum* multilocus microsatellite genotypes identified in brain samples from abortions.

	Microsatellite Genotype ^a^								
Foetus	MS4 (AT)x	MS5 (TA)x	MS6A (TA)x	MS6B (AT)x	MS7 (TA)x	MS8 (AT)x	MS10 (ACT)x − (AGA)y − (TGA)z	MS12 (GT)x	MS21 (TACA)x
**1 ^b^**	14	18	15	12	9.1SEQ	13	6.14.9SEQ	16	10
**2**	14	18			9.1SEQ	13	6.14.9SEQ	16	10
**3**					9.1	**14**	6.14.9	16	
**4 ^b^**	14	18		12	9.1	13	6.14.9	16	10
**5 ^b^**	14	**17**	15	12	9.1	13	6.14.9	16	10
**6**	14		15		9.1		6.14.9SEQ	16	10
**7**		18		12	9.1	13	6.14.9	16	10
**8**	14				9.1	13	6.14.9	16	10
**9 ^b^**	14	18	15	12	9.1	13	6.14.9	16	10
**10**	14	18			9.1	13	6.14.9	16	10
**11**	14	18			9.1	13	6.14.9	16	10
**12 ^b^**	14	18	15	12	9.1	13	6.14.9	16	10
**13 ^b^**	14	18	15	12	9.1	13	6.14.9	16	10
**14 ^b^**	14	18	15		9.1	13	**6.15.9**	16	10
**15**		**17/18**	15				6.14.9	16	10

^a^ The allele polymorphism of microsatellite (MS) markers is expressed as the number of repeats (n and x, y, z for MS10) according to the allele assignment described by [24]. Alleles in bold highlight alleles that differ from the common MS. SEQ indicates MS identification by sequencing in addition to fragment analysis. ^b^ Complete or almost complete microsatellite genotypes.

**Table 2:** Seropositivity and abortion in the dam–offspring pairs.

Dams		Female Offspring	
Presence of Abortion	Serostatus	Presence of Abortion	Serostatus
Abortion (*n* = 84)	Seropositive (*n* = 45)	Abortion (*n* = 25; 55.5% *)	Seropositive (*n* = 22; 88%)
			Seronegative (*n* = 3; 12%)
		No abortion (*n* = 20; 44.5%)	Seropositive (*n* = 19; 95%)
			Seronegative (*n* = 1; 5%)
	Seronegative (*n* = 39)	Abortion (*n* = 6; 15.4% *)	Seropositive (*n* = 0; 0%)
			Seronegative (*n* = 6; 100%)
		No abortion (*n* = 33; 84.6%)	Seropositive (*n* = 1; 3%)
			Seronegative (*n* = 32; 97%)
No abortion (*n* = 161)	Seropositive (*n* = 29)	Abortion (*n* = 9; 31%)	Seropositive (*n* = 9; 100%)
			Seronegative (*n* = 0; 0%)
		No abortion (*n* = 20; 69%)	Seropositive (*n* = 14; 70%)
			Seronegative (*n* = 6; 30%)
	Seronegative (*n* = 132)	Abortion (*n* = 28; 21.2%)	Seropositive (*n* = 2; 7%)
			Seronegative (*n* = 26; 93%)
		No abortion (*n* = 104; 78.8%)	Seropositive (*n* = 4; 3.8%)
			Seronegative (*n* = 100; 96.2%)

* Statistically significant differences between the percentage of aborted offspring born from seropositive and aborted dams and from seronegative and aborted dams (*p* < 0.0001).

**Table 3:** Flock reproductive data prior to and during control programme.

	Year	Number of Female Adult Sheep	Number of Aborted Sheep (% Abortion Rate)	Number of Culled Animals (% Culling Rate)	Number of Replaced Animals (% Replacement Rate)
Prior to the control programme	2011	678	55 (8.1%)	76 (11.2%)	95 (14%)
	2012	697	91 (13.1%)	199 (28.6%)	212 (30.4%)
	2013	710	159 (22.4%)	142 (20%)	210 (29.6%)
	2014	778	146 (18.8%)	228 (29.3%)	271 (34.8%)
	2015	821	131 (16%)	173 (21.1%)	178 (21.7%)
	2016	826	164 (19.9%)	152 (18.4%)	181 (21.9%)
	2017	855	141 (16.5%)	300 (35.1%)	307 (35.9%)
	2018	862	221 (25.6%)	350 (40.6%)	300 (34.8%)
During the control programme	2019	812	132 (16.3%)	266 (32.8%)	373 (46%)
	2020	919	66 (7.2%)	208 (22.6%)	299 (32.5%)

**Table 4:** Collection of samples and diagnostic techniques employed.

Sampling Date	Number of Sampled Animals	Samples	Techniques Employed (Number of Samples Analysed)
March–May 2018	986 female sheep	Serum	NcSALUVET ELISA (all samples)
			NcSALUVET avidity ELISA (in 8 seropositive aborted animals and in 6 seropositive non-aborted animals)
			TgSALUVET ELISA (in 160 aborted animals)
March 2018	2 dogs	Serum	IFAT (all samples)
During 2018	50 aborted sheep	Brains of 39 aborted foetuses (from 34 sheep) and 16 placentas (cotyledons) from 16 sheep were analysed	*N. caninum* PCR (all samples)
			Microsatellite genotyping (in 15 *N. caninum* PCR-positive foetal brains)
			*T. gondii* PCR (all samples)
			Histology (HE staining) (all samples)
From June 2018 to December 2020 (control program)	972 female lambs (one month of age) born from *N. caninum* seronegative dams. Of them, 278 were resampled at 7–10 months of age.	Serum	NcSALUVET ELISA (all samples)

## Data Availability

Not applicable.

## Supplementary Materials

The following are available online at https://www.mdpi.com/article/10.3390/pathogens10111518/s1, Table S1: Receiver operating characteristic (ROC) analysis for NcSALUVET ELISA.
