# Supplementary material for: Dynamics of Neospora caninum-Associated Abortions in a Dairy Sheep Flock and Results of a Test-and-Cull Control Programme
Source: Pathogens. 2021 Nov 20;10(11):1518. doi: 10.3390/pathogens10111518 (PMC8625302; doi:10.3390/pathogens10111518)
Supplement: Supplementary file 1 [file pathogens-10-01518-s001.zip › pathogens-1428798-supplementary/Table S1.pdf]

Table S1. Receiver operating characteristic (ROC) analysis for Nc SALUVET ELISA. The commercial ELISA ID Screen® *Neospora caninum* Indirect (IDvet, Grabels, France) and Western Blotting were used as reference methods. An optimal cut-off of RIPC = 35.08 was selected (bold text), with sensitivity and specificity of 97.73% and 97.30%, respectively. RIPC = relative index per cent; CI = confidence interval; LR + = Positive Likelihood ratio; LR - = Negative Likelihood ratio.

Cutoff (RIPC)	Sensitivity	95% CI	Specificity	95% CI	LR +	LR -
-6.143	1	0.9882 to 1.000	0.00208	0.00005253 to 0.01150	1.0021	0
-5.595	1	0.9882 to 1.000	0.00415	0.000503 to 0.01491	1.0042	0
-4.954	1	0.9882 to 1.000	0.00830	0.002266 to 0.02111	1.0084	0
-4.741	1	0.9882 to 1.000	0.0104	0.003377 to 0.02404	1.0105	0
-4.661	1	0.9882 to 1.000	0.0125	0.004582 to 0.02690	1.0126	0
-4.517	1	0.9882 to 1.000	0.0166	0.007192 to 0.03244	1.0169	0
-4.373	1	0.9882 to 1.000	0.0208	0.009993 to 0.03782	1.0212	0
-4.212	1	0.9882 to 1.000	0.0228	0.01145 to 0.04047	1.0234	0
-3.857	1	0.9882 to 1.000	0.0249	0.01293 to 0.04308	1.0255	0
-3.49	1	0.9882 to 1.000	0.027	0.01444 to 0.04568	1.0277	0
-3.316	1	0.9882 to 1.000	0.0311	0.01752 to 0.05081	1.0321	0
-3.22	1	0.9882 to 1.000	0.0353	0.02068 to 0.05587	1.0366	0
-3.153	1	0.9882 to 1.000	0.0394	0.02390 to 0.06088	1.041	0
-3.009	1	0.9882 to 1.000	0.0415	0.02553 to 0.06336	1.0433	0
-2.835	1	0.9882 to 1.000	0.0456	0.02882 to 0.06829	1.0478	0
-2.739	1	0.9882 to 1.000	0.0477	0.03049 to 0.07074	1.0501	0
-2.643	1	0.9882 to 1.000	0.0498	0.03216 to 0.07318	1.0524	0
-2.594	1	0.9882 to 1.000	0.0539	0.03554 to 0.07804	1.057	0
-2.546	1	0.9882 to 1.000	0.056	0.03724 to 0.08046	1.0593	0
-2.355	1	0.9882 to 1.000	0.0581	0.03894 to 0.08287	1.0617	0
-2.162	1	0.9882 to 1.000	0.0602	0.04066 to 0.08527	1.064	0
-1.97	1	0.9882 to 1.000	0.0622	0.04238 to 0.08766	1.0664	0
-1.805	1	0.9882 to 1.000	0.0685	0.04759 to 0.09481	1.0735	0
-1.565	1	0.9882 to 1.000	0.0705	0.04934 to 0.09718	1.0759	0
-1.298	1	0.9882 to 1.000	0.0726	0.05110 to 0.09954	1.0783	0
-1.201	1	0.9882 to 1.000	0.0768	0.05462 to 0.1043	1.0831	0
-1.105	1	0.9882 to 1.000	0.0788	0.05639 to 0.1066	1.0856	0
-1.027	1	0.9882 to 1.000	0.083	0.05995 to 0.1113	1.0905	0
-0.8829	1	0.9882 to 1.000	0.0851	0.06173 to 0.1136	1.093	0
-0.6728	1	0.9882 to 1.000	0.0871	0.06352 to 0.1160	1.0955	0
-0.5286	1	0.9882 to 1.000	0.0913	0.06711 to 0.1206	1.1005	0
-0.4325	1	0.9882 to 1.000	0.0934	0.06891 to 0.1229	1.103	0
-0.3364	1	0.9882 to 1.000	0.0954	0.07072 to 0.1252	1.1055	0
-0.2403	1	0.9882 to 1.000	0.0975	0.07253 to 0.1276	1.108	0
-0.0481	1	0.9882 to 1.000	0.0996	0.07434 to 0.1299	1.1106	0

0.1204	1	0.9882 to 1.000	0.1017	0.07616 to 0.1322	1.1132	0
0.172	1	0.9882 to 1.000	0.1037	0.07798 to 0.1345	1.1157	0
0.2919	1	0.9882 to 1.000	0.1058	0.07980 to 0.1368	1.1183	0
0.5661	1	0.9882 to 1.000	0.1079	0.08163 to 0.1391	1.121	0
0.7583	1	0.9882 to 1.000	0.11	0.08346 to 0.1414	1.1236	0
0.8425	1	0.9882 to 1.000	0.1141	0.08713 to 0.1459	1.1288	0
0.9866	1	0.9882 to 1.000	0.1162	0.08897 to 0.1482	1.1315	0
1.108	1	0.9882 to 1.000	0.1203	0.09265 to 0.1528	1.1368	0
1.252	1	0.9882 to 1.000	0.1224	0.09450 to 0.1550	1.1395	0
1.372	1	0.9882 to 1.000	0.1266	0.09820 to 0.1596	1.145	0
1.42	1	0.9882 to 1.000	0.1286	0.1001 to 0.1618	1.1476	0
1.49	1	0.9882 to 1.000	0.1307	0.1019 to 0.1641	1.1504	0
1.586	1	0.9882 to 1.000	0.1328	0.1038 to 0.1664	1.1531	0
1.648	1	0.9882 to 1.000	0.1349	0.1056 to 0.1686	1.1559	0
1.734	1	0.9882 to 1.000	0.1369	0.1075 to 0.1709	1.1586	0
1.912	1	0.9882 to 1.000	0.139	0.1094 to 0.1731	1.1614	0
2.163	1	0.9882 to 1.000	0.1411	0.1112 to 0.1754	1.1643	0
2.325	1	0.9882 to 1.000	0.1432	0.1131 to 0.1776	1.1671	0
2.469	1	0.9882 to 1.000	0.1452	0.1150 to 0.1799	1.1699	0
2.672	1	0.9882 to 1.000	0.1473	0.1169 to 0.1821	1.1727	0
2.816	1	0.9882 to 1.000	0.1494	0.1188 to 0.1844	1.1756	0
2.987	1	0.9882 to 1.000	0.1515	0.1206 to 0.1866	1.1786	0
3.257	1	0.9882 to 1.000	0.1535	0.1225 to 0.1889	1.1813	0
3.805	1	0.9882 to 1.000	0.1556	0.1244 to 0.1911	1.1843	0
4.208	1	0.9882 to 1.000	0.1577	0.1263 to 0.1933	1.1872	0
4.332	1	0.9882 to 1.000	0.1598	0.1282 to 0.1956	1.1902	0
4.618	1	0.9882 to 1.000	0.1618	0.1301 to 0.1978	1.193	0
4.85	1	0.9882 to 1.000	0.1639	0.1320 to 0.2000	1.196	0
4.95	1	0.9882 to 1.000	0.166	0.1339 to 0.2023	1.199	0
5.094	1	0.9882 to 1.000	0.168	0.1358 to 0.2045	1.2019	0
5.225	1	0.9882 to 1.000	0.1701	0.1377 to 0.2067	1.205	0
5.369	1	0.9882 to 1.000	0.1722	0.1396 to 0.2089	1.208	0
5.508	1	0.9882 to 1.000	0.1743	0.1415 to 0.2111	1.2111	0
5.541	0.9968	0.9822 to 0.9999	0.1743	0.1415 to 0.2111	1.2072	0.0184
5.583	0.9968	0.9822 to 0.9999	0.1763	0.1434 to 0.2134	1.2101	0.0182
5.646	0.9968	0.9822 to 0.9999	0.1784	0.1453 to 0.2156	1.2132	0.0179
5.778	0.9968	0.9822 to 0.9999	0.1805	0.1472 to 0.2178	1.2164	0.0177
6.018	0.9968	0.9822 to 0.9999	0.1826	0.1491 to 0.2200	1.2195	0.0175
6.224	0.9968	0.9822 to 0.9999	0.1846	0.1510 to 0.2222	1.2225	0.0173
6.355	0.9968	0.9822 to 0.9999	0.1867	0.1529 to 0.2244	1.2256	0.0171
6.509	0.9968	0.9822 to 0.9999	0.1888	0.1548 to 0.2266	1.2288	0.0169
6.825	0.9968	0.9822 to 0.9999	0.1909	0.1567 to 0.2288	1.232	0.0168
7.274	0.9968	0.9822 to 0.9999	0.1929	0.1587 to 0.2311	1.235	0.0166
7.657	0.9968	0.9822 to 0.9999	0.195	0.1606 to 0.2333	1.2383	0.0164
7.921	0.9968	0.9822 to 0.9999	0.1971	0.1625 to 0.2355	1.2415	0.0162

8.104	0.9968	0.9822 to 0.9999	0.2012	0.1663 to 0.2399	1.2479	0.0159
8.288	0.9968	0.9822 to 0.9999	0.2033	0.1683 to 0.2421	1.2512	0.0157
8.421	0.9968	0.9822 to 0.9999	0.2054	0.1702 to 0.2443	1.2545	0.0156
8.446	0.9968	0.9822 to 0.9999	0.2075	0.1721 to 0.2465	1.2578	0.0154
8.529	0.9968	0.9822 to 0.9999	0.2095	0.1740 to 0.2486	1.261	0.0153
8.719	0.9968	0.9822 to 0.9999	0.2116	0.1760 to 0.2508	1.2643	0.0151
8.835	0.9968	0.9822 to 0.9999	0.2137	0.1779 to 0.2530	1.2677	0.015
8.933	0.9968	0.9822 to 0.9999	0.2178	0.1818 to 0.2574	1.2744	0.0147
9.066	0.9968	0.9822 to 0.9999	0.2199	0.1837 to 0.2596	1.2778	0.0146
9.159	0.9968	0.9822 to 0.9999	0.222	0.1857 to 0.2618	1.2812	0.0144
9.226	0.9968	0.9822 to 0.9999	0.2241	0.1876 to 0.2640	1.2847	0.0143
9.274	0.9968	0.9822 to 0.9999	0.2261	0.1895 to 0.2661	1.288	0.0142
9.354	0.9968	0.9822 to 0.9999	0.2282	0.1915 to 0.2683	1.2915	0.014
9.554	0.9968	0.9822 to 0.9999	0.2303	0.1934 to 0.2705	1.2951	0.0139
9.749	0.9968	0.9822 to 0.9999	0.2324	0.1954 to 0.2727	1.2986	0.0138
9.862	0.9968	0.9822 to 0.9999	0.2344	0.1973 to 0.2749	1.302	0.0137
9.936	0.9968	0.9822 to 0.9999	0.2365	0.1993 to 0.2770	1.3056	0.0135
9.977	0.9968	0.9822 to 0.9999	0.2386	0.2012 to 0.2792	1.3092	0.0134
10.03	0.9968	0.9822 to 0.9999	0.2427	0.2051 to 0.2836	1.3163	0.0132
10.06	0.9968	0.9822 to 0.9999	0.2448	0.2071 to 0.2857	1.3199	0.0131
10.17	0.9968	0.9822 to 0.9999	0.2469	0.2090 to 0.2879	1.3236	0.013
10.33	0.9968	0.9822 to 0.9999	0.249	0.2110 to 0.2901	1.3273	0.0129
10.42	0.9968	0.9822 to 0.9999	0.251	0.2129 to 0.2922	1.3308	0.0127
10.5	0.9968	0.9822 to 0.9999	0.2531	0.2149 to 0.2944	1.3346	0.0126
10.57	0.9968	0.9822 to 0.9999	0.2573	0.2188 to 0.2987	1.3421	0.0124
10.62	0.9968	0.9822 to 0.9999	0.2593	0.2208 to 0.3009	1.3458	0.0123
10.66	0.9968	0.9822 to 0.9999	0.2614	0.2227 to 0.3031	1.3496	0.0122
10.69	0.9968	0.9822 to 0.9999	0.2635	0.2247 to 0.3052	1.3534	0.0121
10.71	0.9968	0.9822 to 0.9999	0.2656	0.2266 to 0.3074	1.3573	0.012
10.72	0.9968	0.9822 to 0.9999	0.2676	0.2286 to 0.3095	1.361	0.012
10.74	0.9968	0.9822 to 0.9999	0.2697	0.2306 to 0.3117	1.3649	0.0119
10.8	0.9968	0.9822 to 0.9999	0.2718	0.2325 to 0.3139	1.3689	0.0118
10.9	0.9968	0.9822 to 0.9999	0.2739	0.2345 to 0.3160	1.3728	0.0117
10.97	0.9968	0.9822 to 0.9999	0.2759	0.2365 to 0.3182	1.3766	0.0116
11	0.9968	0.9822 to 0.9999	0.278	0.2384 to 0.3203	1.3806	0.0115
11.02	0.9968	0.9822 to 0.9999	0.2801	0.2404 to 0.3225	1.3846	0.0114
11.06	0.9968	0.9822 to 0.9999	0.2822	0.2424 to 0.3246	1.3887	0.0113
11.07	0.9968	0.9822 to 0.9999	0.2842	0.2444 to 0.3268	1.3926	0.0113
11.12	0.9968	0.9822 to 0.9999	0.2863	0.2463 to 0.3289	1.3967	0.0112
11.19	0.9968	0.9822 to 0.9999	0.2884	0.2483 to 0.3311	1.4008	0.0111
11.32	0.9968	0.9822 to 0.9999	0.2905	0.2503 to 0.3332	1.4049	0.011
11.42	0.9968	0.9822 to 0.9999	0.2925	0.2523 to 0.3354	1.4089	0.0109
11.47	0.9968	0.9822 to 0.9999	0.2946	0.2542 to 0.3375	1.4131	0.0109
11.51	0.9968	0.9822 to 0.9999	0.2967	0.2562 to 0.3397	1.4173	0.0108
11.53	0.9968	0.9822 to 0.9999	0.2988	0.2582 to 0.3418	1.4216	0.0107

11.57	0.9968	0.9822 to 0.9999	0.3008	0.2602 to 0.3439	1.4256	0.0106
11.62	0.9968	0.9822 to 0.9999	0.3029	0.2622 to 0.3461	1.4299	0.0106
11.66	0.9968	0.9822 to 0.9999	0.305	0.2641 to 0.3482	1.4342	0.0105
11.67	0.9968	0.9822 to 0.9999	0.3071	0.2661 to 0.3504	1.4386	0.0104
11.69	0.9968	0.9822 to 0.9999	0.3091	0.2681 to 0.3525	1.4428	0.0104
11.74	0.9968	0.9822 to 0.9999	0.3112	0.2701 to 0.3546	1.4472	0.0103
11.76	0.9968	0.9822 to 0.9999	0.3133	0.2721 to 0.3568	1.4516	0.0102
11.8	0.9968	0.9822 to 0.9999	0.3154	0.2741 to 0.3589	1.456	0.0101
11.83	0.9968	0.9822 to 0.9999	0.3174	0.2761 to 0.3610	1.4603	0.0101
11.84	0.9968	0.9822 to 0.9999	0.3195	0.2781 to 0.3632	1.4648	0.01
11.84	0.9968	0.9822 to 0.9999	0.3216	0.2800 to 0.3653	1.4693	0.01
11.88	0.9968	0.9822 to 0.9999	0.3237	0.2820 to 0.3674	1.4739	0.00990
11.96	0.9968	0.9822 to 0.9999	0.3257	0.2840 to 0.3696	1.4783	0.00980
12.03	0.9968	0.9822 to 0.9999	0.3278	0.2860 to 0.3717	1.4829	0.00980
12.07	0.9968	0.9822 to 0.9999	0.3299	0.2880 to 0.3738	1.4875	0.00970
12.09	0.9968	0.9822 to 0.9999	0.332	0.2900 to 0.3760	1.4922	0.00960
12.1	0.9968	0.9822 to 0.9999	0.334	0.2920 to 0.3781	1.4967	0.00960
12.12	0.9968	0.9822 to 0.9999	0.3361	0.2940 to 0.3802	1.5014	0.00950
12.15	0.9968	0.9822 to 0.9999	0.3382	0.2960 to 0.3823	1.5062	0.00950
12.25	0.9968	0.9822 to 0.9999	0.3402	0.2980 to 0.3845	1.5108	0.00940
12.34	0.9968	0.9822 to 0.9999	0.3423	0.3000 to 0.3866	1.5156	0.00930
12.37	0.9968	0.9822 to 0.9999	0.3444	0.3020 to 0.3887	1.5204	0.00930
12.41	0.9968	0.9822 to 0.9999	0.3465	0.3040 to 0.3908	1.5253	0.00920
12.43	0.9968	0.9822 to 0.9999	0.3485	0.3060 to 0.3930	1.53	0.00920
12.49	0.9968	0.9822 to 0.9999	0.3506	0.3080 to 0.3951	1.535	0.00910
12.55	0.9968	0.9822 to 0.9999	0.3527	0.3100 to 0.3972	1.5399	0.00910
12.57	0.9968	0.9822 to 0.9999	0.3548	0.3120 to 0.3993	1.5449	0.00900
12.6	0.9968	0.9822 to 0.9999	0.3568	0.3140 to 0.4014	1.5498	0.00900
12.63	0.9968	0.9822 to 0.9999	0.3589	0.3160 to 0.4035	1.5548	0.00890
12.67	0.9968	0.9822 to 0.9999	0.361	0.3180 to 0.4057	1.5599	0.00890
12.7	0.9968	0.9822 to 0.9999	0.3631	0.3201 to 0.4078	1.5651	0.00880
12.71	0.9968	0.9822 to 0.9999	0.3651	0.3221 to 0.4099	1.57	0.00880
12.73	0.9968	0.9822 to 0.9999	0.3672	0.3241 to 0.4120	1.5752	0.00870
12.77	0.9968	0.9822 to 0.9999	0.3693	0.3261 to 0.4141	1.5805	0.00870
12.82	0.9968	0.9822 to 0.9999	0.3714	0.3281 to 0.4162	1.5857	0.00860
12.89	0.9968	0.9822 to 0.9999	0.3734	0.3301 to 0.4183	1.5908	0.00860
12.95	0.9968	0.9822 to 0.9999	0.3755	0.3321 to 0.4204	1.5962	0.00850
12.96	0.9968	0.9822 to 0.9999	0.3776	0.3341 to 0.4226	1.6015	0.00850
13.01	0.9968	0.9822 to 0.9999	0.3797	0.3362 to 0.4247	1.607	0.00840
13.05	0.9968	0.9822 to 0.9999	0.3817	0.3382 to 0.4268	1.6122	0.00840
13.06	0.9968	0.9822 to 0.9999	0.3838	0.3402 to 0.4289	1.6177	0.00830
13.12	0.9968	0.9822 to 0.9999	0.3859	0.3422 to 0.4310	1.6232	0.00830
13.25	0.9968	0.9822 to 0.9999	0.388	0.3442 to 0.4331	1.6288	0.00820
13.35	0.9968	0.9822 to 0.9999	0.39	0.3463 to 0.4352	1.6341	0.00820
13.37	0.9968	0.9822 to 0.9999	0.3921	0.3483 to 0.4373	1.6397	0.00820

13.45	0.9968	0.9822 to 0.9999	0.3942	0.3503 to 0.4394	1.6454	0.00810
13.59	0.9968	0.9822 to 0.9999	0.3963	0.3523 to 0.4415	1.6512	0.00810
13.66	0.9968	0.9822 to 0.9999	0.3983	0.3543 to 0.4436	1.6566	0.00800
13.67	0.9968	0.9822 to 0.9999	0.4025	0.3584 to 0.4478	1.6683	0.00800
13.68	0.9968	0.9822 to 0.9999	0.4046	0.3604 to 0.4499	1.6742	0.00790
13.71	0.9968	0.9822 to 0.9999	0.4066	0.3624 to 0.4520	1.6798	0.00790
13.75	0.9968	0.9822 to 0.9999	0.4087	0.3645 to 0.4541	1.6858	0.00780
13.78	0.9968	0.9822 to 0.9999	0.4108	0.3665 to 0.4562	1.6918	0.00780
13.81	0.9968	0.9822 to 0.9999	0.4129	0.3685 to 0.4583	1.6978	0.00780
13.83	0.9968	0.9822 to 0.9999	0.4149	0.3706 to 0.4604	1.7036	0.00770
13.88	0.9968	0.9822 to 0.9999	0.417	0.3726 to 0.4625	1.7098	0.00770
13.96	0.9968	0.9822 to 0.9999	0.4191	0.3746 to 0.4646	1.716	0.00760
14.04	0.9968	0.9822 to 0.9999	0.4212	0.3766 to 0.4666	1.7222	0.00760
14.1	0.9968	0.9822 to 0.9999	0.4253	0.3807 to 0.4708	1.7345	0.00750
14.13	0.9968	0.9822 to 0.9999	0.4274	0.3827 to 0.4729	1.7408	0.00750
14.14	0.9968	0.9822 to 0.9999	0.4315	0.3868 to 0.4771	1.7534	0.00740
14.15	0.9968	0.9822 to 0.9999	0.4336	0.3889 to 0.4792	1.7599	0.00740
14.2	0.9968	0.9822 to 0.9999	0.4357	0.3909 to 0.4813	1.7664	0.00730
14.23	0.9968	0.9822 to 0.9999	0.4378	0.3929 to 0.4834	1.773	0.00730
14.25	0.9968	0.9822 to 0.9999	0.4398	0.3950 to 0.4854	1.7794	0.00730
14.34	0.9968	0.9822 to 0.9999	0.4419	0.3970 to 0.4875	1.7861	0.00720
14.42	0.9968	0.9822 to 0.9999	0.444	0.3991 to 0.4896	1.7928	0.00720
14.45	0.9968	0.9822 to 0.9999	0.4461	0.4011 to 0.4917	1.7996	0.00720
14.48	0.9968	0.9822 to 0.9999	0.4481	0.4031 to 0.4938	1.8061	0.00710
14.53	0.9968	0.9822 to 0.9999	0.4502	0.4052 to 0.4959	1.813	0.00710
14.57	0.9968	0.9822 to 0.9999	0.4523	0.4072 to 0.4979	1.82	0.00710
14.57	0.9968	0.9822 to 0.9999	0.4544	0.4093 to 0.5000	1.827	0.00700
14.58	0.9968	0.9822 to 0.9999	0.4564	0.4113 to 0.5021	1.8337	0.00700
14.65	0.9968	0.9822 to 0.9999	0.4585	0.4134 to 0.5042	1.8408	0.00700
14.72	0.9968	0.9822 to 0.9999	0.4606	0.4154 to 0.5062	1.848	0.00690
14.75	0.9968	0.9822 to 0.9999	0.4627	0.4175 to 0.5083	1.8552	0.00690
14.84	0.9968	0.9822 to 0.9999	0.4647	0.4195 to 0.5104	1.8621	0.00690
14.96	0.9968	0.9822 to 0.9999	0.4668	0.4216 to 0.5125	1.8695	0.00690
15.04	0.9968	0.9822 to 0.9999	0.4689	0.4236 to 0.5145	1.8769	0.00680
15.1	0.9968	0.9822 to 0.9999	0.471	0.4257 to 0.5166	1.8843	0.00680
15.14	0.9968	0.9822 to 0.9999	0.473	0.4277 to 0.5187	1.8915	0.00680
15.16	0.9968	0.9822 to 0.9999	0.4751	0.4298 to 0.5208	1.899	0.00670
15.19	0.9968	0.9822 to 0.9999	0.4772	0.4318 to 0.5228	1.9067	0.00670
15.2	0.9968	0.9822 to 0.9999	0.4793	0.4339 to 0.5249	1.9143	0.00670
15.22	0.9968	0.9822 to 0.9999	0.4834	0.4380 to 0.5290	1.9295	0.00660
15.24	0.9968	0.9822 to 0.9999	0.4855	0.4400 to 0.5311	1.9374	0.00660
15.28	0.9968	0.9822 to 0.9999	0.4876	0.4421 to 0.5332	1.9454	0.00660
15.34	0.9968	0.9822 to 0.9999	0.4896	0.4441 to 0.5352	1.953	0.00650
15.37	0.9968	0.9822 to 0.9999	0.4917	0.4462 to 0.5373	1.961	0.00650
15.41	0.9968	0.9822 to 0.9999	0.4938	0.4483 to 0.5394	1.9692	0.00650

15.52	0.9968	0.9822 to 0.9999	0.4959	0.4503 to 0.5414	1.9774	0.00650
15.64	0.9968	0.9822 to 0.9999	0.4979	0.4524 to 0.5435	1.9853	0.00640
15.7	0.9968	0.9822 to 0.9999	0.5	0.4544 to 0.5456	1.9936	0.00640
15.72	0.9968	0.9822 to 0.9999	0.5021	0.4565 to 0.5476	2.002	0.00640
15.74	0.9968	0.9822 to 0.9999	0.5041	0.4586 to 0.5497	2.0101	0.00630
15.8	0.9968	0.9822 to 0.9999	0.5062	0.4606 to 0.5517	2.0186	0.00630
15.89	0.9968	0.9822 to 0.9999	0.5083	0.4627 to 0.5538	2.0273	0.00630
15.94	0.9968	0.9822 to 0.9999	0.5104	0.4648 to 0.5559	2.0359	0.00630
15.98	0.9968	0.9822 to 0.9999	0.5124	0.4668 to 0.5579	2.0443	0.00620
16.04	0.9968	0.9822 to 0.9999	0.5145	0.4689 to 0.5600	2.0531	0.00620
16.06	0.9968	0.9822 to 0.9999	0.5187	0.4730 to 0.5641	2.0711	0.00620
16.1	0.9968	0.9822 to 0.9999	0.5207	0.4751 to 0.5661	2.0797	0.00610
16.15	0.9968	0.9822 to 0.9999	0.5228	0.4772 to 0.5682	2.0889	0.00610
16.17	0.9968	0.9822 to 0.9999	0.5249	0.4792 to 0.5702	2.0981	0.00610
16.2	0.9968	0.9822 to 0.9999	0.527	0.4813 to 0.5723	2.1074	0.00610
16.24	0.9968	0.9822 to 0.9999	0.529	0.4834 to 0.5743	2.1163	0.00600
16.26	0.9968	0.9822 to 0.9999	0.5311	0.4855 to 0.5764	2.1258	0.00600
16.3	0.9968	0.9822 to 0.9999	0.5332	0.4875 to 0.5784	2.1354	0.00600
16.34	0.9968	0.9822 to 0.9999	0.5353	0.4896 to 0.5805	2.145	0.00600
16.35	0.9968	0.9822 to 0.9999	0.5373	0.4917 to 0.5825	2.1543	0.00600
16.37	0.9968	0.9822 to 0.9999	0.5394	0.4938 to 0.5846	2.1641	0.00590
16.4	0.9968	0.9822 to 0.9999	0.5415	0.4958 to 0.5866	2.174	0.00590
16.42	0.9968	0.9822 to 0.9999	0.5436	0.4979 to 0.5887	2.184	0.00590
16.44	0.9968	0.9822 to 0.9999	0.5456	0.5000 to 0.5907	2.1937	0.00590
16.48	0.9968	0.9822 to 0.9999	0.5477	0.5021 to 0.5928	2.2038	0.00580
16.51	0.9968	0.9822 to 0.9999	0.5498	0.5041 to 0.5948	2.2141	0.00580
16.57	0.9968	0.9822 to 0.9999	0.5519	0.5062 to 0.5969	2.2245	0.00580
16.62	0.9968	0.9822 to 0.9999	0.5539	0.5083 to 0.5989	2.2345	0.00580
16.66	0.9968	0.9822 to 0.9999	0.556	0.5104 to 0.6009	2.245	0.00580
16.7	0.9968	0.9822 to 0.9999	0.5581	0.5125 to 0.6030	2.2557	0.00570
16.71	0.9968	0.9822 to 0.9999	0.5602	0.5146 to 0.6050	2.2665	0.00570
16.79	0.9968	0.9822 to 0.9999	0.5622	0.5166 to 0.6071	2.2768	0.00570
16.88	0.9968	0.9822 to 0.9999	0.5643	0.5187 to 0.6091	2.2878	0.00570
16.89	0.9968	0.9822 to 0.9999	0.5664	0.5208 to 0.6111	2.2989	0.00560
16.93	0.9968	0.9822 to 0.9999	0.5685	0.5229 to 0.6132	2.3101	0.00560
16.97	0.9968	0.9822 to 0.9999	0.5705	0.5250 to 0.6152	2.3208	0.00560
17.01	0.9968	0.9822 to 0.9999	0.5726	0.5271 to 0.6173	2.3322	0.00560
17.09	0.9968	0.9822 to 0.9999	0.5747	0.5292 to 0.6193	2.3438	0.00560
17.14	0.9968	0.9822 to 0.9999	0.5768	0.5313 to 0.6213	2.3554	0.00550
17.17	0.9968	0.9822 to 0.9999	0.5788	0.5334 to 0.6234	2.3666	0.00550
17.2	0.9968	0.9822 to 0.9999	0.5809	0.5354 to 0.6254	2.3784	0.00550
17.24	0.9968	0.9822 to 0.9999	0.5851	0.5396 to 0.6294	2.4025	0.00550
17.26	0.9968	0.9822 to 0.9999	0.5871	0.5417 to 0.6315	2.4141	0.00550
17.32	0.9968	0.9822 to 0.9999	0.5892	0.5438 to 0.6335	2.4265	0.00540
17.41	0.9968	0.9822 to 0.9999	0.5913	0.5459 to 0.6355	2.439	0.00540

17.48	0.9968	0.9822 to 0.9999	0.5934	0.5480 to 0.6376	2.4515	0.00540
17.53	0.9968	0.9822 to 0.9999	0.5954	0.5501 to 0.6396	2.4637	0.00540
17.57	0.9968	0.9822 to 0.9999	0.5975	0.5522 to 0.6416	2.4765	0.00540
17.62	0.9968	0.9822 to 0.9999	0.5996	0.5543 to 0.6436	2.4895	0.00530
17.67	0.9968	0.9822 to 0.9999	0.6017	0.5564 to 0.6457	2.5026	0.00530
17.71	0.9968	0.9822 to 0.9999	0.6037	0.5585 to 0.6477	2.5153	0.00530
17.75	0.9968	0.9822 to 0.9999	0.6058	0.5606 to 0.6497	2.5287	0.00530
17.81	0.9968	0.9822 to 0.9999	0.6079	0.5627 to 0.6517	2.5422	0.00530
17.84	0.9968	0.9822 to 0.9999	0.61	0.5648 to 0.6537	2.5559	0.00520
17.87	0.9968	0.9822 to 0.9999	0.612	0.5669 to 0.6558	2.5691	0.00520
17.88	0.9968	0.9822 to 0.9999	0.6141	0.5690 to 0.6578	2.5831	0.00520
17.91	0.9968	0.9822 to 0.9999	0.6162	0.5711 to 0.6598	2.5972	0.00520
17.97	0.9968	0.9822 to 0.9999	0.6183	0.5732 to 0.6618	2.6115	0.00520
18.01	0.9968	0.9822 to 0.9999	0.6203	0.5753 to 0.6638	2.6252	0.00520
18.03	0.9968	0.9822 to 0.9999	0.6224	0.5774 to 0.6659	2.6398	0.00510
18.05	0.9968	0.9822 to 0.9999	0.6245	0.5796 to 0.6679	2.6546	0.00510
18.07	0.9968	0.9822 to 0.9999	0.6266	0.5817 to 0.6699	2.6695	0.00510
18.09	0.9968	0.9822 to 0.9999	0.6286	0.5838 to 0.6719	2.6839	0.00510
18.12	0.9968	0.9822 to 0.9999	0.6307	0.5859 to 0.6739	2.6992	0.00510
18.15	0.9968	0.9822 to 0.9999	0.6328	0.5880 to 0.6759	2.7146	0.00510
18.17	0.9968	0.9822 to 0.9999	0.6349	0.5901 to 0.6779	2.7302	0.00500
18.2	0.9968	0.9822 to 0.9999	0.6369	0.5922 to 0.6799	2.7452	0.00500
18.24	0.9968	0.9822 to 0.9999	0.639	0.5943 to 0.6820	2.7612	0.00500
18.29	0.9968	0.9822 to 0.9999	0.6411	0.5965 to 0.6840	2.7774	0.00500
18.34	0.9968	0.9822 to 0.9999	0.6432	0.5986 to 0.6860	2.7937	0.00500
18.39	0.9968	0.9822 to 0.9999	0.6452	0.6007 to 0.6880	2.8095	0.00500
18.46	0.9968	0.9822 to 0.9999	0.6473	0.6028 to 0.6900	2.8262	0.00490
18.54	0.9968	0.9822 to 0.9999	0.6494	0.6049 to 0.6920	2.8431	0.00490
18.56	0.9968	0.9822 to 0.9999	0.6515	0.6070 to 0.6940	2.8603	0.00490
18.57	0.9968	0.9822 to 0.9999	0.6535	0.6092 to 0.6960	2.8768	0.00490
18.59	0.9968	0.9822 to 0.9999	0.6556	0.6113 to 0.6980	2.8943	0.00490
18.63	0.9968	0.9822 to 0.9999	0.6598	0.6155 to 0.7020	2.93	0.00480
18.73	0.9968	0.9822 to 0.9999	0.6618	0.6177 to 0.7040	2.9474	0.00480
18.85	0.9968	0.9822 to 0.9999	0.6639	0.6198 to 0.7060	2.9658	0.00480
18.9	0.9968	0.9822 to 0.9999	0.666	0.6219 to 0.7080	2.9844	0.00480
18.91	0.9968	0.9822 to 0.9999	0.668	0.6240 to 0.7100	3.0024	0.00480
18.92	0.9968	0.9822 to 0.9999	0.6701	0.6262 to 0.7120	3.0215	0.00480
18.96	0.9968	0.9822 to 0.9999	0.6722	0.6283 to 0.7140	3.0409	0.00480
18.99	0.9968	0.9822 to 0.9999	0.6743	0.6304 to 0.7160	3.0605	0.00470
19.05	0.9968	0.9822 to 0.9999	0.6763	0.6326 to 0.7180	3.0794	0.00470
19.22	0.9968	0.9822 to 0.9999	0.6784	0.6347 to 0.7200	3.0995	0.00470
19.36	0.9968	0.9822 to 0.9999	0.6805	0.6368 to 0.7219	3.1199	0.00470
19.4	0.9968	0.9822 to 0.9999	0.6826	0.6390 to 0.7239	3.1405	0.00470
19.49	0.9968	0.9822 to 0.9999	0.6846	0.6411 to 0.7259	3.1604	0.00470
19.62	0.9968	0.9822 to 0.9999	0.6867	0.6432 to 0.7279	3.1816	0.00470

19.7	0.9968	0.9822 to 0.9999	0.6888	0.6454 to 0.7299	3.2031	0.00460
19.78	0.9968	0.9822 to 0.9999	0.6909	0.6475 to 0.7319	3.2248	0.00460
19.85	0.9968	0.9822 to 0.9999	0.6929	0.6496 to 0.7339	3.2458	0.00460
19.88	0.9968	0.9822 to 0.9999	0.695	0.6518 to 0.7359	3.2682	0.00460
19.91	0.9968	0.9822 to 0.9999	0.6971	0.6539 to 0.7378	3.2909	0.00460
19.93	0.9968	0.9822 to 0.9999	0.6992	0.6561 to 0.7398	3.3138	0.00460
20.04	0.9968	0.9822 to 0.9999	0.7012	0.6582 to 0.7418	3.336	0.00460
20.12	0.9968	0.9822 to 0.9999	0.7033	0.6603 to 0.7438	3.3596	0.00450
20.15	0.9968	0.9822 to 0.9999	0.7054	0.6625 to 0.7458	3.3836	0.00450
20.19	0.9968	0.9822 to 0.9999	0.7075	0.6646 to 0.7477	3.4079	0.00450
20.2	0.9968	0.9822 to 0.9999	0.7095	0.6668 to 0.7497	3.4313	0.00450
20.28	0.9935	0.9769 to 0.9992	0.7095	0.6668 to 0.7497	3.42	0.00920
20.35	0.9935	0.9769 to 0.9992	0.7137	0.6711 to 0.7537	3.4701	0.00910
20.36	0.9935	0.9769 to 0.9992	0.7158	0.6732 to 0.7556	3.4958	0.00910
20.4	0.9935	0.9769 to 0.9992	0.7178	0.6754 to 0.7576	3.5206	0.00910
20.48	0.9935	0.9769 to 0.9992	0.7199	0.6775 to 0.7596	3.5469	0.00900
20.68	0.9935	0.9769 to 0.9992	0.722	0.6797 to 0.7616	3.5737	0.00900
20.88	0.9935	0.9769 to 0.9992	0.7241	0.6818 to 0.7635	3.6009	0.00900
20.94	0.9935	0.9769 to 0.9992	0.7261	0.6840 to 0.7655	3.6272	0.00900
20.95	0.9935	0.9769 to 0.9992	0.7282	0.6861 to 0.7675	3.6553	0.00890
20.96	0.9935	0.9769 to 0.9992	0.7303	0.6883 to 0.7694	3.6837	0.00890
20.98	0.9935	0.9769 to 0.9992	0.7344	0.6926 to 0.7734	3.7406	0.00890
21	0.9935	0.9769 to 0.9992	0.7386	0.6969 to 0.7773	3.8007	0.00880
21.1	0.9935	0.9769 to 0.9992	0.7407	0.6991 to 0.7792	3.8315	0.00880
21.21	0.9935	0.9769 to 0.9992	0.7427	0.7013 to 0.7812	3.8613	0.00880
21.22	0.9935	0.9769 to 0.9992	0.7448	0.7034 to 0.7832	3.893	0.00870
21.32	0.9935	0.9769 to 0.9992	0.7469	0.7056 to 0.7851	3.9253	0.00870
21.44	0.9935	0.9769 to 0.9992	0.749	0.7078 to 0.7871	3.9582	0.00870
21.54	0.9935	0.9769 to 0.9992	0.751	0.7099 to 0.7890	3.99	0.00870
21.68	0.9935	0.9769 to 0.9992	0.7531	0.7121 to 0.7910	4.0239	0.00860
21.76	0.9935	0.9769 to 0.9992	0.7552	0.7143 to 0.7929	4.0584	0.00860
21.82	0.9935	0.9769 to 0.9992	0.7573	0.7164 to 0.7949	4.0935	0.00860
21.87	0.9935	0.9769 to 0.9992	0.7593	0.7186 to 0.7968	4.1275	0.00860
21.9	0.9935	0.9769 to 0.9992	0.7614	0.7208 to 0.7988	4.1639	0.00850
21.96	0.9935	0.9769 to 0.9992	0.7635	0.7230 to 0.8007	4.2008	0.00850
22	0.9935	0.9769 to 0.9992	0.7676	0.7273 to 0.8046	4.275	0.00850
22.08	0.9935	0.9769 to 0.9992	0.7697	0.7295 to 0.8066	4.3139	0.00840
22.15	0.9935	0.9769 to 0.9992	0.7718	0.7317 to 0.8085	4.3536	0.00840
22.17	0.9935	0.9769 to 0.9992	0.7739	0.7339 to 0.8105	4.3941	0.00840
22.21	0.9935	0.9769 to 0.9992	0.7759	0.7360 to 0.8124	4.4333	0.00840
22.23	0.9935	0.9769 to 0.9992	0.778	0.7382 to 0.8143	4.4752	0.00840
22.28	0.9935	0.9769 to 0.9992	0.7801	0.7404 to 0.8163	4.518	0.00830
22.33	0.9935	0.9769 to 0.9992	0.7822	0.7426 to 0.8182	4.5615	0.00830
22.35	0.9935	0.9769 to 0.9992	0.7842	0.7448 to 0.8202	4.6038	0.00830
22.47	0.9935	0.9769 to 0.9992	0.7863	0.7470 to 0.8221	4.649	0.00830

22.6	0.9935	0.9769 to 0.9992	0.7884	0.7492 to 0.8240	4.6952	0.00820
22.72	0.9935	0.9769 to 0.9992	0.7905	0.7514 to 0.8260	4.7422	0.00820
22.9	0.9935	0.9769 to 0.9992	0.7925	0.7535 to 0.8279	4.788	0.00820
23.06	0.9935	0.9769 to 0.9992	0.7946	0.7557 to 0.8298	4.8369	0.00820
23.2	0.9935	0.9769 to 0.9992	0.7988	0.7601 to 0.8337	4.9379	0.00810
23.27	0.9935	0.9769 to 0.9992	0.8008	0.7623 to 0.8356	4.9874	0.00810
23.34	0.9935	0.9769 to 0.9992	0.8029	0.7645 to 0.8375	5.0406	0.00810
23.41	0.9935	0.9769 to 0.9992	0.805	0.7667 to 0.8394	5.0949	0.00810
23.45	0.9935	0.9769 to 0.9992	0.8071	0.7689 to 0.8413	5.1503	0.00810
23.53	0.9935	0.9769 to 0.9992	0.8091	0.7712 to 0.8433	5.2043	0.00800
23.6	0.9935	0.9769 to 0.9992	0.8112	0.7734 to 0.8452	5.2622	0.00800
23.64	0.9935	0.9769 to 0.9992	0.8133	0.7756 to 0.8471	5.3214	0.00800
23.7	0.9935	0.9769 to 0.9992	0.8154	0.7778 to 0.8490	5.3819	0.00800
23.74	0.9935	0.9769 to 0.9992	0.8174	0.7800 to 0.8509	5.4409	0.00800
23.79	0.9935	0.9769 to 0.9992	0.8195	0.7822 to 0.8528	5.5042	0.00790
23.87	0.9935	0.9769 to 0.9992	0.8216	0.7844 to 0.8547	5.5689	0.00790
24	0.9935	0.9769 to 0.9992	0.8237	0.7866 to 0.8566	5.6353	0.00790
24.08	0.9935	0.9769 to 0.9992	0.8257	0.7889 to 0.8585	5.6999	0.00790
24.13	0.9935	0.9769 to 0.9992	0.8278	0.7911 to 0.8604	5.7695	0.00790
24.18	0.9935	0.9769 to 0.9992	0.832	0.7955 to 0.8642	5.9137	0.00780
24.21	0.9935	0.9769 to 0.9992	0.834	0.7977 to 0.8661	5.9849	0.00780
24.38	0.9935	0.9769 to 0.9992	0.8361	0.8000 to 0.8680	6.0616	0.00780
24.58	0.9935	0.9769 to 0.9992	0.8382	0.8022 to 0.8699	6.1403	0.00780
24.73	0.9935	0.9769 to 0.9992	0.8402	0.8044 to 0.8718	6.2171	0.00770
24.88	0.9935	0.9769 to 0.9992	0.8423	0.8067 to 0.8737	6.2999	0.00770
24.96	0.9935	0.9769 to 0.9992	0.8444	0.8089 to 0.8756	6.385	0.00770
25.05	0.9935	0.9769 to 0.9992	0.8465	0.8111 to 0.8775	6.4723	0.00770
25.15	0.9935	0.9769 to 0.9992	0.8485	0.8134 to 0.8794	6.5578	0.00770
25.26	0.9935	0.9769 to 0.9992	0.8506	0.8156 to 0.8812	6.6499	0.00760
25.43	0.9935	0.9769 to 0.9992	0.8527	0.8179 to 0.8831	6.7447	0.00760
25.55	0.9903	0.9720 to 0.9980	0.8527	0.8179 to 0.8831	6.723	0.0114
25.65	0.9903	0.9720 to 0.9980	0.8548	0.8201 to 0.8850	6.8202	0.0113
25.8	0.9903	0.9720 to 0.9980	0.8568	0.8224 to 0.8869	6.9155	0.0113
25.9	0.9903	0.9720 to 0.9980	0.8589	0.8246 to 0.8888	7.0184	0.0113
25.92	0.9903	0.9720 to 0.9980	0.861	0.8269 to 0.8906	7.1245	0.0113
25.94	0.9903	0.9720 to 0.9980	0.8631	0.8291 to 0.8925	7.2337	0.0112
25.98	0.9903	0.9720 to 0.9980	0.8651	0.8314 to 0.8944	7.341	0.0112
26.1	0.9903	0.9720 to 0.9980	0.8672	0.8336 to 0.8962	7.4571	0.0112
26.21	0.9903	0.9720 to 0.9980	0.8693	0.8359 to 0.8981	7.5769	0.0112
26.27	0.9903	0.9720 to 0.9980	0.8714	0.8382 to 0.8999	7.7006	0.0111
26.46	0.9903	0.9720 to 0.9980	0.8734	0.8404 to 0.9018	7.8223	0.0111
26.84	0.9903	0.9720 to 0.9980	0.8755	0.8427 to 0.9036	7.9542	0.0111
27.09	0.9903	0.9720 to 0.9980	0.8776	0.8450 to 0.9055	8.0907	0.0111
27.12	0.9903	0.9720 to 0.9980	0.8797	0.8472 to 0.9073	8.2319	0.011
27.28	0.9903	0.9720 to 0.9980	0.8817	0.8495 to 0.9092	8.3711	0.011

27.44	0.9903	0.9720 to 0.9980	0.8838	0.8518 to 0.9110	8.5224	0.011
27.51	0.9903	0.9720 to 0.9980	0.8859	0.8541 to 0.9129	8.6792	0.0109
27.64	0.9871	0.9673 to 0.9965	0.8859	0.8541 to 0.9129	8.6512	0.0146
27.77	0.9871	0.9673 to 0.9965	0.89	0.8586 to 0.9165	8.9736	0.0145
27.82	0.9871	0.9673 to 0.9965	0.8921	0.8609 to 0.9184	9.1483	0.0145
27.94	0.9871	0.9673 to 0.9965	0.8942	0.8632 to 0.9202	9.3299	0.0144
28.03	0.9871	0.9673 to 0.9965	0.8963	0.8655 to 0.9220	9.5188	0.0144
28.13	0.9871	0.9673 to 0.9965	0.8983	0.8678 to 0.9238	9.706	0.0144
28.23	0.9871	0.9673 to 0.9965	0.9004	0.8701 to 0.9257	9.9106	0.0143
28.4	0.9871	0.9673 to 0.9965	0.9025	0.8724 to 0.9275	10.1241	0.0143
28.72	0.9871	0.9673 to 0.9965	0.9046	0.8748 to 0.9293	10.347	0.0143
28.88	0.9871	0.9673 to 0.9965	0.9066	0.8771 to 0.9311	10.5685	0.0142
28.89	0.9871	0.9673 to 0.9965	0.9087	0.8794 to 0.9329	10.8116	0.0142
28.93	0.9871	0.9673 to 0.9965	0.9108	0.8817 to 0.9347	11.0661	0.0142
29.03	0.9871	0.9673 to 0.9965	0.9129	0.8840 to 0.9365	11.333	0.0141
29.12	0.9871	0.9673 to 0.9965	0.9149	0.8864 to 0.9383	11.5993	0.0141
29.22	0.9871	0.9673 to 0.9965	0.917	0.8887 to 0.9401	11.8928	0.0141
29.41	0.9838	0.9628 to 0.9947	0.917	0.8887 to 0.9401	11.853	0.0177
29.53	0.9838	0.9628 to 0.9947	0.9191	0.8910 to 0.9418	12.1607	0.0176
29.6	0.9838	0.9628 to 0.9947	0.9212	0.8934 to 0.9436	12.4848	0.0176
29.66	0.9838	0.9628 to 0.9947	0.9232	0.8957 to 0.9454	12.8099	0.0175
29.73	0.9838	0.9628 to 0.9947	0.9253	0.8981 to 0.9471	13.17	0.0175
29.84	0.9838	0.9628 to 0.9947	0.9274	0.9005 to 0.9489	13.551	0.0175
29.89	0.9838	0.9628 to 0.9947	0.9295	0.9028 to 0.9507	13.9546	0.0174
29.97	0.9838	0.9628 to 0.9947	0.9315	0.9052 to 0.9524	14.362	0.0174
30.05	0.9838	0.9628 to 0.9947	0.9336	0.9076 to 0.9541	14.8163	0.0174
30.31	0.9838	0.9628 to 0.9947	0.9357	0.9099 to 0.9559	15.3002	0.0173
30.58	0.9838	0.9628 to 0.9947	0.9378	0.9123 to 0.9576	15.8167	0.0173
31.02	0.9838	0.9628 to 0.9947	0.9398	0.9147 to 0.9593	16.3422	0.0172
31.66	0.9838	0.9628 to 0.9947	0.9419	0.9171 to 0.9611	16.9329	0.0172
31.95	0.9838	0.9628 to 0.9947	0.944	0.9195 to 0.9628	17.5679	0.0172
32	0.9838	0.9628 to 0.9947	0.9461	0.9220 to 0.9645	18.2523	0.0171
32.04	0.9838	0.9628 to 0.9947	0.9481	0.9244 to 0.9662	18.9557	0.0171
32.12	0.9838	0.9628 to 0.9947	0.9502	0.9268 to 0.9678	19.755	0.017
32.31	0.9838	0.9628 to 0.9947	0.9523	0.9293 to 0.9695	20.6247	0.017
32.59	0.9838	0.9628 to 0.9947	0.9544	0.9317 to 0.9712	21.5746	0.017
32.73	0.9838	0.9628 to 0.9947	0.9564	0.9342 to 0.9728	22.5642	0.0169
32.8	0.9838	0.9628 to 0.9947	0.9585	0.9366 to 0.9745	23.706	0.0169
33.02	0.9806	0.9584 to 0.9929	0.9585	0.9366 to 0.9745	23.6289	0.0202
33.23	0.9806	0.9584 to 0.9929	0.9606	0.9391 to 0.9761	24.8883	0.0202
33.33	0.9806	0.9584 to 0.9929	0.9627	0.9416 to 0.9777	26.2895	0.0202
33.43	0.9806	0.9584 to 0.9929	0.9647	0.9441 to 0.9793	27.779	0.0201
33.69	0.9806	0.9584 to 0.9929	0.9668	0.9467 to 0.9809	29.5361	0.0201
34.15	0.9806	0.9584 to 0.9929	0.9689	0.9492 to 0.9825	31.5305	0.02
34.64	0.9806	0.9584 to 0.9929	0.971	0.9517 to 0.9840	33.8138	0.02

34.96	0.9806	0.9584 to 0.9929	0.973	0.9543 to 0.9856	36.3185	0.0199
**35.08**	**0.9773**	**0.9540 to 0.9909**	**0.973**	**0.9543 to 0.9856**	**36.1963**	**0.0233**
35.15	0.9773	0.9540 to 0.9909	0.9751	0.9569 to 0.9871	39.249	0.0233
35.31	0.9773	0.9540 to 0.9909	0.9772	0.9595 to 0.9886	42.864	0.0232
35.55	0.9773	0.9540 to 0.9909	0.9793	0.9622 to 0.9900	47.2126	0.0232
35.91	0.9773	0.9540 to 0.9909	0.9813	0.9649 to 0.9914	52.262	0.0231
36.35	0.9741	0.9498 to 0.9888	0.9813	0.9649 to 0.9914	52.0909	0.0264
36.62	0.9709	0.9456 to 0.9866	0.9813	0.9649 to 0.9914	51.9198	0.0297
37.04	0.9709	0.9456 to 0.9866	0.9834	0.9676 to 0.9928	58.488	0.0296
37.49	0.9676	0.9415 to 0.9844	0.9834	0.9676 to 0.9928	58.2892	0.0329
37.62	0.9676	0.9415 to 0.9844	0.9855	0.9703 to 0.9941	66.731	0.0329
37.69	0.9676	0.9415 to 0.9844	0.9876	0.9731 to 0.9954	78.0323	0.0328
37.9	0.9676	0.9415 to 0.9844	0.9896	0.9760 to 0.9966	93.0385	0.0327
38.17	0.9644	0.9374 to 0.9822	0.9896	0.9760 to 0.9966	92.7308	0.036
38.32	0.9644	0.9374 to 0.9822	0.9917	0.9789 to 0.9977	116.1928	0.0359
38.42	0.9644	0.9374 to 0.9822	0.9938	0.9819 to 0.9987	155.5484	0.0358
39.01	0.9612	0.9334 to 0.9798	0.9938	0.9819 to 0.9987	155.0323	0.039
39.58	0.9579	0.9294 to 0.9775	0.9938	0.9819 to 0.9987	154.5	0.0424
40.08	0.9547	0.9254 to 0.9751	0.9938	0.9819 to 0.9987	153.9839	0.0456
40.94	0.9515	0.9214 to 0.9727	0.9938	0.9819 to 0.9987	153.4677	0.0488
41.67	0.9515	0.9214 to 0.9727	0.9959	0.9851 to 0.9995	232.0732	0.0487
42.19	0.9482	0.9175 to 0.9702	0.9959	0.9851 to 0.9995	231.2683	0.052
42.92	0.945	0.9136 to 0.9677	0.9959	0.9851 to 0.9995	230.4878	0.0552
43.93	0.9417	0.9098 to 0.9652	0.9959	0.9851 to 0.9995	229.6829	0.0585
44.95	0.9385	0.9059 to 0.9627	0.9959	0.9851 to 0.9995	228.9024	0.0618
45.53	0.9353	0.9021 to 0.9601	0.9959	0.9851 to 0.9995	228.122	0.065
45.74	0.932	0.8983 to 0.9576	0.9959	0.9851 to 0.9995	227.3171	0.0683
46.98	0.9288	0.8945 to 0.9550	0.9959	0.9851 to 0.9995	226.5366	0.0715
48.39	0.9256	0.8908 to 0.9524	0.9959	0.9851 to 0.9995	225.7561	0.0747
49.14	0.9223	0.8870 to 0.9498	0.9959	0.9851 to 0.9995	224.9512	0.078
49.57	0.9191	0.8833 to 0.9471	0.9959	0.9851 to 0.9995	224.1707	0.0812
50.17	0.9159	0.8795 to 0.9445	0.9959	0.9851 to 0.9995	223.3902	0.0844
51.27	0.9159	0.8795 to 0.9445	0.9979	0.9885 to 0.9999	436.1429	0.0843
51.78	0.9126	0.8758 to 0.9418	0.9979	0.9885 to 0.9999	434.5714	0.0876
51.92	0.9094	0.8721 to 0.9391	0.9979	0.9885 to 0.9999	433.0476	0.0908
53.03	0.9094	0.8721 to 0.9391	1	0.9924 to 1.000		0.0906
54.12	0.9061	0.8684 to 0.9365	1	0.9924 to 1.000		0.0939
56.7	0.9029	0.8647 to 0.9338	1	0.9924 to 1.000		0.0971
59.21	0.8997	0.8611 to 0.9310	1	0.9924 to 1.000		0.1003
59.4	0.8964	0.8574 to 0.9283	1	0.9924 to 1.000		0.1036
59.71	0.8932	0.8538 to 0.9256	1	0.9924 to 1.000		0.1068
60.43	0.89	0.8501 to 0.9228	1	0.9924 to 1.000		0.11
61	0.8867	0.8465 to 0.9201	1	0.9924 to 1.000		0.1133
61.53	0.8835	0.8429 to 0.9173	1	0.9924 to 1.000		0.1165
62.12	0.8803	0.8393 to 0.9146	1	0.9924 to 1.000		0.1197

62.21	0.877	0.8356 to 0.9118	1	0.9924 to 1.000	0.123
62.67	0.8738	0.8321 to 0.9090	1	0.9924 to 1.000	0.1262
63.15	0.8706	0.8285 to 0.9062	1	0.9924 to 1.000	0.1294
63.23	0.8673	0.8249 to 0.9034	1	0.9924 to 1.000	0.1327
63.24	0.8641	0.8213 to 0.9006	1	0.9924 to 1.000	0.1359
63.58	0.8608	0.8177 to 0.8978	1	0.9924 to 1.000	0.1392
64.29	0.8576	0.8142 to 0.8949	1	0.9924 to 1.000	0.1424
64.93	0.8544	0.8106 to 0.8921	1	0.9924 to 1.000	0.1456
65.21	0.8511	0.8071 to 0.8893	1	0.9924 to 1.000	0.1489
65.4	0.8479	0.8035 to 0.8864	1	0.9924 to 1.000	0.1521
65.62	0.8447	0.8000 to 0.8836	1	0.9924 to 1.000	0.1553
65.83	0.8414	0.7965 to 0.8807	1	0.9924 to 1.000	0.1586
66.35	0.8382	0.7929 to 0.8779	1	0.9924 to 1.000	0.1618
66.83	0.835	0.7894 to 0.8750	1	0.9924 to 1.000	0.165
67.17	0.8317	0.7859 to 0.8721	1	0.9924 to 1.000	0.1683
67.79	0.8285	0.7824 to 0.8692	1	0.9924 to 1.000	0.1715
68.8	0.8252	0.7789 to 0.8663	1	0.9924 to 1.000	0.1748
69.63	0.822	0.7754 to 0.8635	1	0.9924 to 1.000	0.178
69.89	0.8188	0.7719 to 0.8606	1	0.9924 to 1.000	0.1812
70.08	0.8123	0.7650 to 0.8548	1	0.9924 to 1.000	0.1877
70.28	0.8091	0.7615 to 0.8518	1	0.9924 to 1.000	0.1909
70.56	0.8058	0.7580 to 0.8489	1	0.9924 to 1.000	0.1942
70.78	0.8026	0.7545 to 0.8460	1	0.9924 to 1.000	0.1974
71.12	0.7994	0.7511 to 0.8431	1	0.9924 to 1.000	0.2006
71.46	0.7961	0.7476 to 0.8402	1	0.9924 to 1.000	0.2039
72.45	0.7929	0.7442 to 0.8372	1	0.9924 to 1.000	0.2071
73.95	0.7896	0.7407 to 0.8343	1	0.9924 to 1.000	0.2104
74.51	0.7864	0.7373 to 0.8313	1	0.9924 to 1.000	0.2136
74.7	0.7832	0.7338 to 0.8284	1	0.9924 to 1.000	0.2168
74.98	0.7799	0.7304 to 0.8254	1	0.9924 to 1.000	0.2201
75.11	0.7767	0.7270 to 0.8225	1	0.9924 to 1.000	0.2233
75.23	0.7735	0.7235 to 0.8195	1	0.9924 to 1.000	0.2265
75.49	0.7702	0.7201 to 0.8166	1	0.9924 to 1.000	0.2298
75.81	0.767	0.7167 to 0.8136	1	0.9924 to 1.000	0.233
76.07	0.7638	0.7133 to 0.8106	1	0.9924 to 1.000	0.2362
76.24	0.7605	0.7099 to 0.8077	1	0.9924 to 1.000	0.2395
76.42	0.7573	0.7064 to 0.8047	1	0.9924 to 1.000	0.2427
76.83	0.754	0.7030 to 0.8017	1	0.9924 to 1.000	0.246
77.45	0.7508	0.6996 to 0.7987	1	0.9924 to 1.000	0.2492
77.9	0.7476	0.6962 to 0.7957	1	0.9924 to 1.000	0.2524
78.23	0.7443	0.6928 to 0.7927	1	0.9924 to 1.000	0.2557
78.64	0.7411	0.6894 to 0.7897	1	0.9924 to 1.000	0.2589
78.9	0.7379	0.6861 to 0.7867	1	0.9924 to 1.000	0.2621
79.43	0.7346	0.6827 to 0.7837	1	0.9924 to 1.000	0.2654
79.89	0.7314	0.6793 to 0.7807	1	0.9924 to 1.000	0.2686

79.98	0.7282	0.6759 to 0.7777	1	0.9924 to 1.000	0.2718
80.12	0.7249	0.6725 to 0.7747	1	0.9924 to 1.000	0.2751
80.24	0.7217	0.6692 to 0.7717	1	0.9924 to 1.000	0.2783
80.63	0.7184	0.6658 to 0.7687	1	0.9924 to 1.000	0.2816
81.05	0.7152	0.6624 to 0.7657	1	0.9924 to 1.000	0.2848
81.33	0.712	0.6591 to 0.7626	1	0.9924 to 1.000	0.288
81.54	0.7087	0.6557 to 0.7596	1	0.9924 to 1.000	0.2913
81.58	0.7055	0.6523 to 0.7566	1	0.9924 to 1.000	0.2945
81.86	0.7023	0.6490 to 0.7535	1	0.9924 to 1.000	0.2977
82.29	0.699	0.6456 to 0.7505	1	0.9924 to 1.000	0.301
82.73	0.6958	0.6423 to 0.7475	1	0.9924 to 1.000	0.3042
83.25	0.6926	0.6389 to 0.7444	1	0.9924 to 1.000	0.3074
83.65	0.6893	0.6356 to 0.7414	1	0.9924 to 1.000	0.3107
83.86	0.6861	0.6323 to 0.7383	1	0.9924 to 1.000	0.3139
84.13	0.6828	0.6289 to 0.7353	1	0.9924 to 1.000	0.3172
84.42	0.6796	0.6256 to 0.7322	1	0.9924 to 1.000	0.3204
84.73	0.6764	0.6223 to 0.7292	1	0.9924 to 1.000	0.3236
84.98	0.6731	0.6189 to 0.7261	1	0.9924 to 1.000	0.3269
85.01	0.6699	0.6156 to 0.7230	1	0.9924 to 1.000	0.3301
85.03	0.6667	0.6123 to 0.7200	1	0.9924 to 1.000	0.3333
85.06	0.6634	0.6090 to 0.7169	1	0.9924 to 1.000	0.3366
85.1	0.6602	0.6056 to 0.7138	1	0.9924 to 1.000	0.3398
85.29	0.657	0.6023 to 0.7107	1	0.9924 to 1.000	0.343
85.5	0.6537	0.5990 to 0.7077	1	0.9924 to 1.000	0.3463
85.58	0.6505	0.5957 to 0.7046	1	0.9924 to 1.000	0.3495
85.62	0.6472	0.5924 to 0.7015	1	0.9924 to 1.000	0.3528
85.67	0.644	0.5891 to 0.6984	1	0.9924 to 1.000	0.356
85.83	0.6408	0.5858 to 0.6953	1	0.9924 to 1.000	0.3592
86.31	0.6375	0.5825 to 0.6922	1	0.9924 to 1.000	0.3625
86.69	0.6343	0.5792 to 0.6892	1	0.9924 to 1.000	0.3657
86.81	0.6311	0.5759 to 0.6861	1	0.9924 to 1.000	0.3689
87.1	0.6278	0.5726 to 0.6830	1	0.9924 to 1.000	0.3722
87.4	0.6246	0.5693 to 0.6799	1	0.9924 to 1.000	0.3754
87.55	0.6214	0.5660 to 0.6768	1	0.9924 to 1.000	0.3786
87.7	0.6181	0.5628 to 0.6736	1	0.9924 to 1.000	0.3819
87.91	0.6149	0.5595 to 0.6705	1	0.9924 to 1.000	0.3851
88.06	0.6117	0.5562 to 0.6674	1	0.9924 to 1.000	0.3883
88.21	0.6084	0.5529 to 0.6643	1	0.9924 to 1.000	0.3916
88.49	0.6052	0.5496 to 0.6612	1	0.9924 to 1.000	0.3948
88.74	0.6019	0.5464 to 0.6581	1	0.9924 to 1.000	0.3981
88.89	0.5987	0.5431 to 0.6550	1	0.9924 to 1.000	0.4013
89.11	0.5955	0.5398 to 0.6518	1	0.9924 to 1.000	0.4045
89.34	0.5922	0.5366 to 0.6487	1	0.9924 to 1.000	0.4078
89.56	0.589	0.5333 to 0.6456	1	0.9924 to 1.000	0.411
89.75	0.5858	0.5301 to 0.6425	1	0.9924 to 1.000	0.4142

89.79	0.5825	0.5268 to 0.6393	1	0.9924 to 1.000	0.4175
89.87	0.5793	0.5235 to 0.6362	1	0.9924 to 1.000	0.4207
90.05	0.5761	0.5203 to 0.6330	1	0.9924 to 1.000	0.4239
90.21	0.5728	0.5170 to 0.6299	1	0.9924 to 1.000	0.4272
90.48	0.5696	0.5138 to 0.6268	1	0.9924 to 1.000	0.4304
90.82	0.5663	0.5106 to 0.6236	1	0.9924 to 1.000	0.4337
91.09	0.5631	0.5073 to 0.6205	1	0.9924 to 1.000	0.4369
91.31	0.5599	0.5041 to 0.6173	1	0.9924 to 1.000	0.4401
91.5	0.5566	0.5008 to 0.6142	1	0.9924 to 1.000	0.4434
91.8	0.5534	0.4976 to 0.6110	1	0.9924 to 1.000	0.4466
92.19	0.5502	0.4944 to 0.6079	1	0.9924 to 1.000	0.4498
92.45	0.5469	0.4911 to 0.6047	1	0.9924 to 1.000	0.4531
92.5	0.5437	0.4879 to 0.6015	1	0.9924 to 1.000	0.4563
92.61	0.5405	0.4847 to 0.5984	1	0.9924 to 1.000	0.4595
92.71	0.5372	0.4815 to 0.5952	1	0.9924 to 1.000	0.4628
92.76	0.534	0.4782 to 0.5920	1	0.9924 to 1.000	0.466
92.9	0.5307	0.4750 to 0.5889	1	0.9924 to 1.000	0.4693
93.02	0.5275	0.4718 to 0.5857	1	0.9924 to 1.000	0.4725
93.05	0.5243	0.4686 to 0.5825	1	0.9924 to 1.000	0.4757
93.14	0.521	0.4654 to 0.5793	1	0.9924 to 1.000	0.479
93.24	0.5178	0.4622 to 0.5762	1	0.9924 to 1.000	0.4822
93.32	0.5146	0.4590 to 0.5730	1	0.9924 to 1.000	0.4854
93.39	0.5113	0.4558 to 0.5698	1	0.9924 to 1.000	0.4887
93.49	0.5081	0.4526 to 0.5666	1	0.9924 to 1.000	0.4919
93.82	0.5049	0.4494 to 0.5634	1	0.9924 to 1.000	0.4951
94.31	0.5016	0.4430 to 0.5570	1	0.9924 to 1.000	0.4984
94.82	0.4984	0.4430 to 0.5570	1	0.9924 to 1.000	0.5016
95.1	0.4951	0.4366 to 0.5506	1	0.9924 to 1.000	0.5049
95.13	0.4919	0.4334 to 0.5474	1	0.9924 to 1.000	0.5081
95.15	0.4887	0.4302 to 0.5442	1	0.9924 to 1.000	0.5113
95.16	0.4854	0.4270 to 0.5410	1	0.9924 to 1.000	0.5146
95.19	0.4822	0.4238 to 0.5378	1	0.9924 to 1.000	0.5178
95.33	0.479	0.4207 to 0.5346	1	0.9924 to 1.000	0.521
95.48	0.4757	0.4175 to 0.5314	1	0.9924 to 1.000	0.5243
95.53	0.4725	0.4143 to 0.5282	1	0.9924 to 1.000	0.5275
95.73	0.4693	0.4111 to 0.5250	1	0.9924 to 1.000	0.5307
96.13	0.466	0.4080 to 0.5218	1	0.9924 to 1.000	0.534
96.4	0.4628	0.4048 to 0.5185	1	0.9924 to 1.000	0.5372
96.53	0.4595	0.4016 to 0.5153	1	0.9924 to 1.000	0.5405
96.68	0.4563	0.3985 to 0.5121	1	0.9924 to 1.000	0.5437
96.8	0.4531	0.3953 to 0.5089	1	0.9924 to 1.000	0.5469
97.01	0.4498	0.3921 to 0.5056	1	0.9924 to 1.000	0.5502
97.32	0.4466	0.3890 to 0.5024	1	0.9924 to 1.000	0.5534
97.53	0.4434	0.3858 to 0.4992	1	0.9924 to 1.000	0.5566
97.64	0.4401	0.3827 to 0.4959	1	0.9924 to 1.000	0.5599

97.76	0.4369	0.3795 to 0.4927	1	0.9924 to 1.000	0.5631
97.95	0.4337	0.3764 to 0.4894	1	0.9924 to 1.000	0.5663
98.07	0.4304	0.3732 to 0.4862	1	0.9924 to 1.000	0.5696
98.3	0.4239	0.3670 to 0.4797	1	0.9924 to 1.000	0.5761
98.5	0.4207	0.3638 to 0.4765	1	0.9924 to 1.000	0.5793
98.7	0.4175	0.3607 to 0.4732	1	0.9924 to 1.000	0.5825
98.92	0.4142	0.3575 to 0.4699	1	0.9924 to 1.000	0.5858
98.97	0.411	0.3544 to 0.4667	1	0.9924 to 1.000	0.589
99.02	0.4078	0.3513 to 0.4634	1	0.9924 to 1.000	0.5922
99.07	0.4045	0.3482 to 0.4602	1	0.9924 to 1.000	0.5955
99.11	0.4013	0.3450 to 0.4569	1	0.9924 to 1.000	0.5987
99.28	0.3981	0.3419 to 0.4536	1	0.9924 to 1.000	0.6019
99.45	0.3948	0.3388 to 0.4504	1	0.9924 to 1.000	0.6052
99.48	0.3916	0.3357 to 0.4471	1	0.9924 to 1.000	0.6084
99.52	0.3883	0.3326 to 0.4438	1	0.9924 to 1.000	0.6117
99.59	0.3851	0.3295 to 0.4405	1	0.9924 to 1.000	0.6149
99.65	0.3819	0.3264 to 0.4372	1	0.9924 to 1.000	0.6181
99.68	0.3786	0.3232 to 0.4340	1	0.9924 to 1.000	0.6214
99.72	0.3754	0.3201 to 0.4307	1	0.9924 to 1.000	0.6246
99.76	0.3722	0.3170 to 0.4274	1	0.9924 to 1.000	0.6278
99.93	0.3689	0.3139 to 0.4241	1	0.9924 to 1.000	0.6311
100.1	0.3657	0.3108 to 0.4208	1	0.9924 to 1.000	0.6343
100.4	0.3625	0.3078 to 0.4175	1	0.9924 to 1.000	0.6375
100.7	0.3592	0.3047 to 0.4142	1	0.9924 to 1.000	0.6408
100.8	0.356	0.3016 to 0.4109	1	0.9924 to 1.000	0.644
100.9	0.3528	0.2985 to 0.4076	1	0.9924 to 1.000	0.6472
101	0.3495	0.2954 to 0.4043	1	0.9924 to 1.000	0.6505
101.1	0.3463	0.2923 to 0.4010	1	0.9924 to 1.000	0.6537
101.1	0.343	0.2893 to 0.3977	1	0.9924 to 1.000	0.657
101.2	0.3398	0.2862 to 0.3944	1	0.9924 to 1.000	0.6602
101.3	0.3366	0.2831 to 0.3910	1	0.9924 to 1.000	0.6634
101.4	0.3333	0.2800 to 0.3877	1	0.9924 to 1.000	0.6667
101.4	0.3301	0.2770 to 0.3844	1	0.9924 to 1.000	0.6699
101.6	0.3269	0.2739 to 0.3811	1	0.9924 to 1.000	0.6731
102	0.3236	0.2708 to 0.3777	1	0.9924 to 1.000	0.6764
102.1	0.3204	0.2678 to 0.3744	1	0.9924 to 1.000	0.6796
102.2	0.3172	0.2647 to 0.3711	1	0.9924 to 1.000	0.6828
102.4	0.3139	0.2617 to 0.3677	1	0.9924 to 1.000	0.6861
102.6	0.3107	0.2586 to 0.3644	1	0.9924 to 1.000	0.6893
102.6	0.3074	0.2556 to 0.3611	1	0.9924 to 1.000	0.6926
102.7	0.3042	0.2525 to 0.3577	1	0.9924 to 1.000	0.6958
102.8	0.301	0.2495 to 0.3544	1	0.9924 to 1.000	0.699
102.9	0.2977	0.2465 to 0.3510	1	0.9924 to 1.000	0.7023
103.2	0.2945	0.2434 to 0.3477	1	0.9924 to 1.000	0.7055
103.4	0.2913	0.2404 to 0.3443	1	0.9924 to 1.000	0.7087

103.6	0.288	0.2374 to 0.3409	1	0.9924 to 1.000	0.712
103.6	0.2848	0.2343 to 0.3376	1	0.9924 to 1.000	0.7152
103.7	0.2783	0.2283 to 0.3308	1	0.9924 to 1.000	0.7217
103.8	0.2751	0.2253 to 0.3275	1	0.9924 to 1.000	0.7249
104.2	0.2718	0.2223 to 0.3241	1	0.9924 to 1.000	0.7282
104.6	0.2686	0.2193 to 0.3207	1	0.9924 to 1.000	0.7314
104.7	0.2654	0.2163 to 0.3173	1	0.9924 to 1.000	0.7346
104.7	0.2621	0.2133 to 0.3139	1	0.9924 to 1.000	0.7379
104.9	0.2589	0.2103 to 0.3106	1	0.9924 to 1.000	0.7411
105.1	0.2557	0.2073 to 0.3072	1	0.9924 to 1.000	0.7443
105.1	0.2524	0.2043 to 0.3038	1	0.9924 to 1.000	0.7476
105.3	0.2492	0.2013 to 0.3004	1	0.9924 to 1.000	0.7508
105.4	0.246	0.1983 to 0.2970	1	0.9924 to 1.000	0.754
105.7	0.2427	0.1953 to 0.2936	1	0.9924 to 1.000	0.7573
106	0.2395	0.1923 to 0.2901	1	0.9924 to 1.000	0.7605
106.2	0.2362	0.1894 to 0.2867	1	0.9924 to 1.000	0.7638
106.3	0.233	0.1864 to 0.2833	1	0.9924 to 1.000	0.767
106.4	0.2298	0.1834 to 0.2799	1	0.9924 to 1.000	0.7702
106.5	0.2265	0.1805 to 0.2765	1	0.9924 to 1.000	0.7735
106.9	0.2233	0.1775 to 0.2730	1	0.9924 to 1.000	0.7767
107.5	0.2201	0.1746 to 0.2696	1	0.9924 to 1.000	0.7799
107.7	0.2168	0.1716 to 0.2662	1	0.9924 to 1.000	0.7832
107.8	0.2136	0.1687 to 0.2627	1	0.9924 to 1.000	0.7864
107.9	0.2104	0.1657 to 0.2593	1	0.9924 to 1.000	0.7896
108	0.2071	0.1628 to 0.2558	1	0.9924 to 1.000	0.7929
108	0.2039	0.1598 to 0.2524	1	0.9924 to 1.000	0.7961
108.1	0.2006	0.1569 to 0.2489	1	0.9924 to 1.000	0.7994
108.5	0.1974	0.1540 to 0.2455	1	0.9924 to 1.000	0.8026
108.8	0.1942	0.1511 to 0.2420	1	0.9924 to 1.000	0.8058
109	0.1909	0.1482 to 0.2385	1	0.9924 to 1.000	0.8091
109.2	0.1877	0.1452 to 0.2350	1	0.9924 to 1.000	0.8123
109.3	0.1845	0.1423 to 0.2316	1	0.9924 to 1.000	0.8155
109.4	0.1812	0.1394 to 0.2281	1	0.9924 to 1.000	0.8188
109.6	0.178	0.1365 to 0.2246	1	0.9924 to 1.000	0.822
109.8	0.1748	0.1337 to 0.2211	1	0.9924 to 1.000	0.8252
110	0.1715	0.1308 to 0.2176	1	0.9924 to 1.000	0.8285
110.3	0.1683	0.1279 to 0.2141	1	0.9924 to 1.000	0.8317
110.6	0.165	0.1250 to 0.2106	1	0.9924 to 1.000	0.835
110.7	0.1618	0.1221 to 0.2071	1	0.9924 to 1.000	0.8382
110.9	0.1586	0.1193 to 0.2035	1	0.9924 to 1.000	0.8414
111.1	0.1553	0.1164 to 0.2000	1	0.9924 to 1.000	0.8447
111.4	0.1521	0.1136 to 0.1965	1	0.9924 to 1.000	0.8479
111.7	0.1489	0.1107 to 0.1929	1	0.9924 to 1.000	0.8511
111.8	0.1456	0.1079 to 0.1894	1	0.9924 to 1.000	0.8544
111.9	0.1424	0.1051 to 0.1858	1	0.9924 to 1.000	0.8576

112	0.1392	0.1022 to 0.1823	1	0.9924 to 1.000	0.8608
112.5	0.1359	0.09942 to 0.1787	1	0.9924 to 1.000	0.8641
113	0.1327	0.09661 to 0.1751	1	0.9924 to 1.000	0.8673
113.1	0.1294	0.09381 to 0.1715	1	0.9924 to 1.000	0.8706
113.4	0.1262	0.09101 to 0.1679	1	0.9924 to 1.000	0.8738
113.9	0.123	0.08822 to 0.1644	1	0.9924 to 1.000	0.877
114.3	0.1197	0.08545 to 0.1607	1	0.9924 to 1.000	0.8803
114.5	0.1165	0.08267 to 0.1571	1	0.9924 to 1.000	0.8835
114.6	0.1133	0.07991 to 0.1535	1	0.9924 to 1.000	0.8867
114.7	0.11	0.07716 to 0.1499	1	0.9924 to 1.000	0.89
115	0.1068	0.07442 to 0.1462	1	0.9924 to 1.000	0.8932
115.2	0.1036	0.07168 to 0.1426	1	0.9924 to 1.000	0.8964
115.3	0.1003	0.06896 to 0.1389	1	0.9924 to 1.000	0.8997
115.4	0.0971	0.06625 to 0.1353	1	0.9924 to 1.000	0.9029
115.6	0.0939	0.06355 to 0.1316	1	0.9924 to 1.000	0.9062
115.9	0.0906	0.06086 to 0.1279	1	0.9924 to 1.000	0.9094
116.1	0.0874	0.05818 to 0.1242	1	0.9924 to 1.000	0.9126
116.4	0.0841	0.05552 to 0.1205	1	0.9924 to 1.000	0.9159
116.5	0.0809	0.05287 to 0.1167	1	0.9924 to 1.000	0.9191
117.3	0.0777	0.05023 to 0.1130	1	0.9924 to 1.000	0.9223
118.5	0.0744	0.04761 to 0.1092	1	0.9924 to 1.000	0.9256
119	0.0712	0.04501 to 0.1055	1	0.9924 to 1.000	0.9288
119.4	0.068	0.04242 to 0.1017	1	0.9924 to 1.000	0.932
119.8	0.0647	0.03985 to 0.09788	1	0.9924 to 1.000	0.9353
120.1	0.0615	0.03730 to 0.09406	1	0.9924 to 1.000	0.9385
120.3	0.0583	0.03477 to 0.09022	1	0.9924 to 1.000	0.9418
120.9	0.055	0.03227 to 0.08635	1	0.9924 to 1.000	0.945
121.5	0.0518	0.02979 to 0.08246	1	0.9924 to 1.000	0.9482
121.8	0.0485	0.02733 to 0.07855	1	0.9924 to 1.000	0.9515
122.3	0.0453	0.02491 to 0.07461	1	0.9924 to 1.000	0.9547
122.9	0.0421	0.02251 to 0.07064	1	0.9924 to 1.000	0.9579
123.4	0.0388	0.02016 to 0.06664	1	0.9924 to 1.000	0.9612
125.2	0.0356	0.01784 to 0.06260	1	0.9924 to 1.000	0.9644
127.3	0.0324	0.01558 to 0.05852	1	0.9924 to 1.000	0.9676
130.5	0.0291	0.01336 to 0.05440	1	0.9924 to 1.000	0.9709
133.3	0.0259	0.01121 to 0.05021	1	0.9924 to 1.000	0.9741
133.7	0.0227	0.009126 to 0.04597	1	0.9924 to 1.000	0.9774
134.6	0.0194	0.007135 to 0.04165	1	0.9924 to 1.000	0.9806
135.2	0.0162	0.005257 to 0.03724	1	0.9924 to 1.000	0.9838
136.1	0.0129	0.003527 to 0.03271	1	0.9924 to 1.000	0.9871
138.5	0.00971	0.002000 to 0.02802	1	0.9924 to 1.000	0.9903
143.3	0.00647	0.000782 to 0.02311	1	0.9924 to 1.000	0.9935
147.6	0.00324	0.00008167 to 0.01784	1	0.9924 to 1.000	0.9968
